# Supplementary material for: Structural and Functional Insights into Bacillus subtilis Sigma Factor Inhibitor, CsfB
Source: Structure. 2018 Apr 3;26(4):640–648.e5. doi: 10.1016/j.str.2018.02.007 (PMC5890618; doi:10.1016/j.str.2018.02.007)
Supplement: Document S1. Figures S1–S5 and Tables S1–S5 [file mmc1.pdf]

Structure, Volume *26*

Supplemental Information

Structural and Functional Insights into *Bacillus subtilis* Sigma Factor Inhibitor, CsfB

Santiago Martínez-Lumbreras, Caterina Alfano, Nicola J. Evans, Katherine M. Collins, Kelly A. Flanagan, R. Andrew Atkinson, Ewelina M. Krysztofinska, Anupama Vydyanath, Jacquelin Jackter, Sarah Fixon-Owoo, Amy H. Camp, and Rivka L. Isaacson

SUPPLEMENTAL FIGURES S1-S4

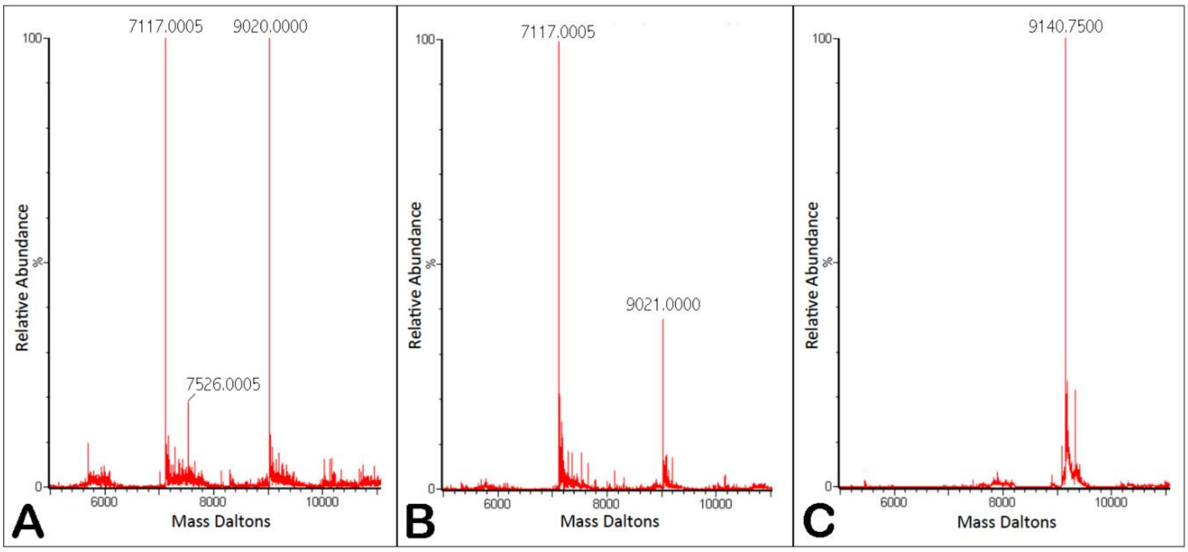

Figure S1 (related to Figure 1): Electrospray ionization mass spectrometry of CsfB. (A) N-terminally His-tagged CsfB immediately following size-exclusion chromatography purification displayed two distinct species with mass correlating to full length (9020.16 Da) and a 1-48 cleaved species (7116.86 Da) at a 50:50 ratio. **(B)** After storage at 4°C for four days the cleaved species represented 75% of the sample. **(C)** The CsfB^{A48E} variant was protected from degradation, remaining uncleaved even after storage at 4°C for four days.

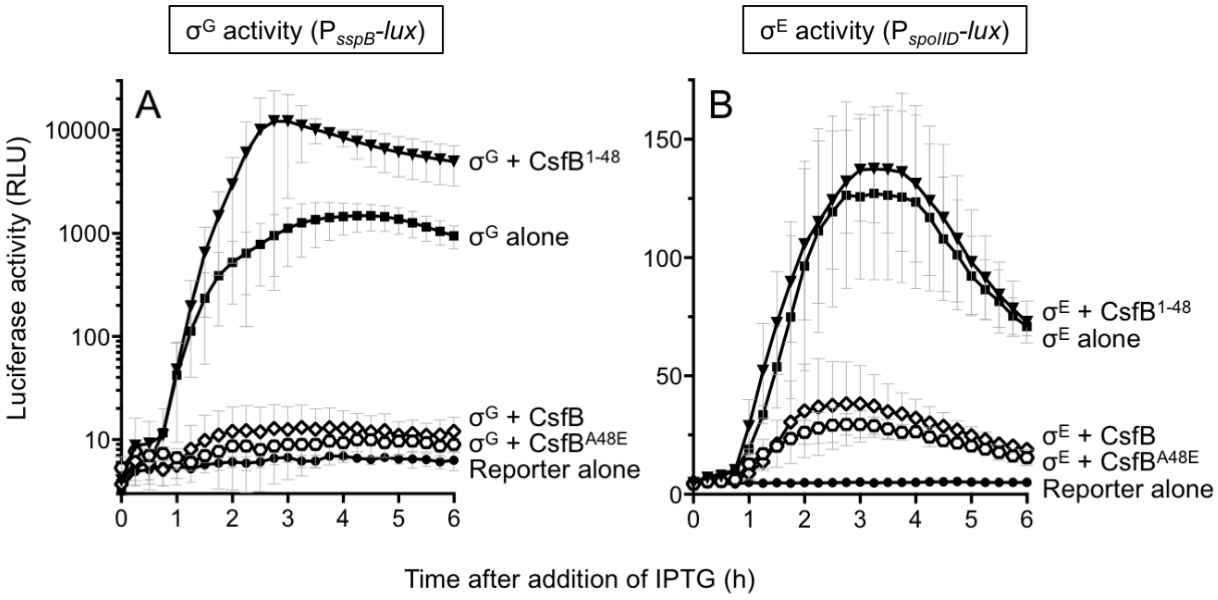

Figure S2 (related to Figure 1): Functionality of the CsfB$^{1\text{-}48}$ and CsfB^{A48E} variants *in vivo*. To assess the ability of CsfB$^{1\text{-}48}$ and CsfB^{A48E} to inhibit **(A)** σ^G or **(B)** σ^E *in vivo*, expression of each sigma factor was induced during vegetative growth either alone or in combination with wild type or variant CsfB proteins. Sigma factor activity following the addition of inducer (IPTG) was monitored every 15 minutes for 6 hours by light production (measured in relative light units [RLU]) from σ^G- or σ^E-dependent luciferase reporter genes (P_{sspB}-*lux* or P_{spoIID}-*lux*, respectively). Light production by strains expressing σ^G or σ^E alone or in combination with wild type CsfB, as well as a control strains without any inducible constructs ("Reporter alone") are shown for comparison in each graph. Data are reported as the average of at least two experiments, with error bars indicating standard deviation. Strains used in this assay are listed in Table S4.

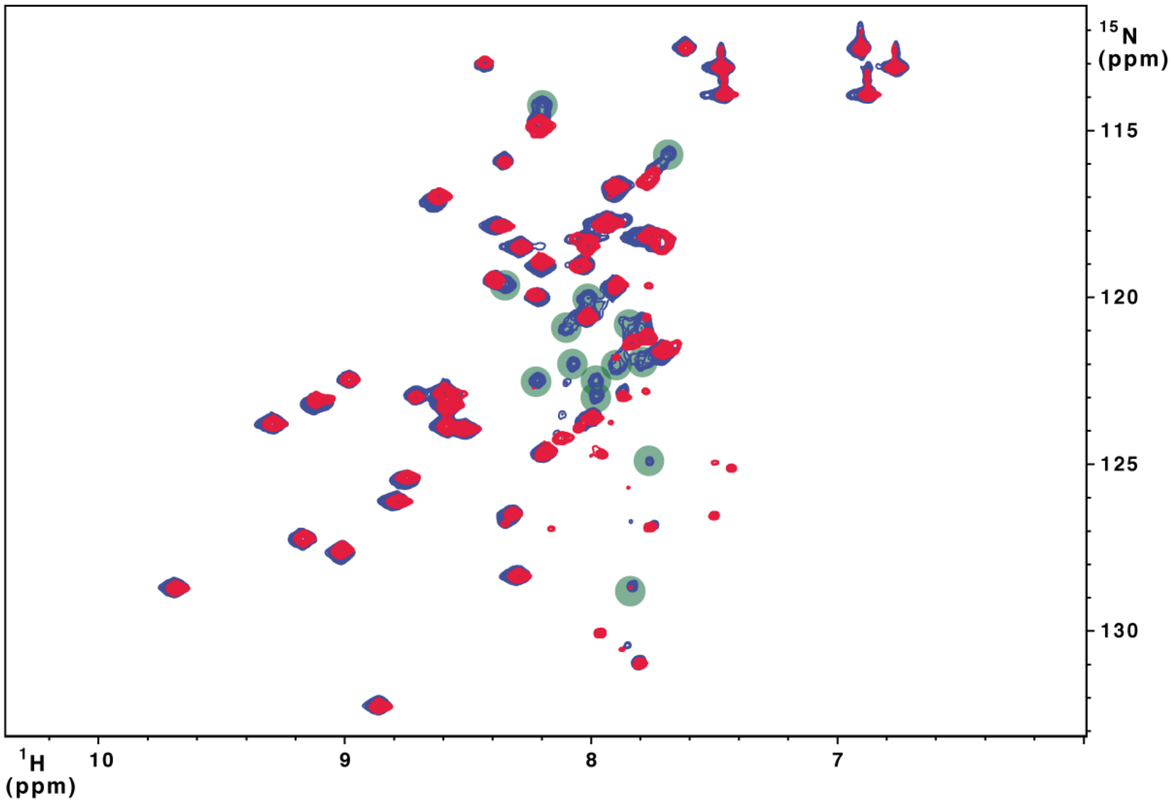

Figure S3 (Related to Figure 1): C-terminally His-tagged CsfB degradation can be observed by NMR spectroscopy. ^{1}H-^{15}N HSQC spectra of fresh protein immediately following purification (blue) and after two days (red). Shadowed peaks correspond to the unstable C-terminal residues of the protein.

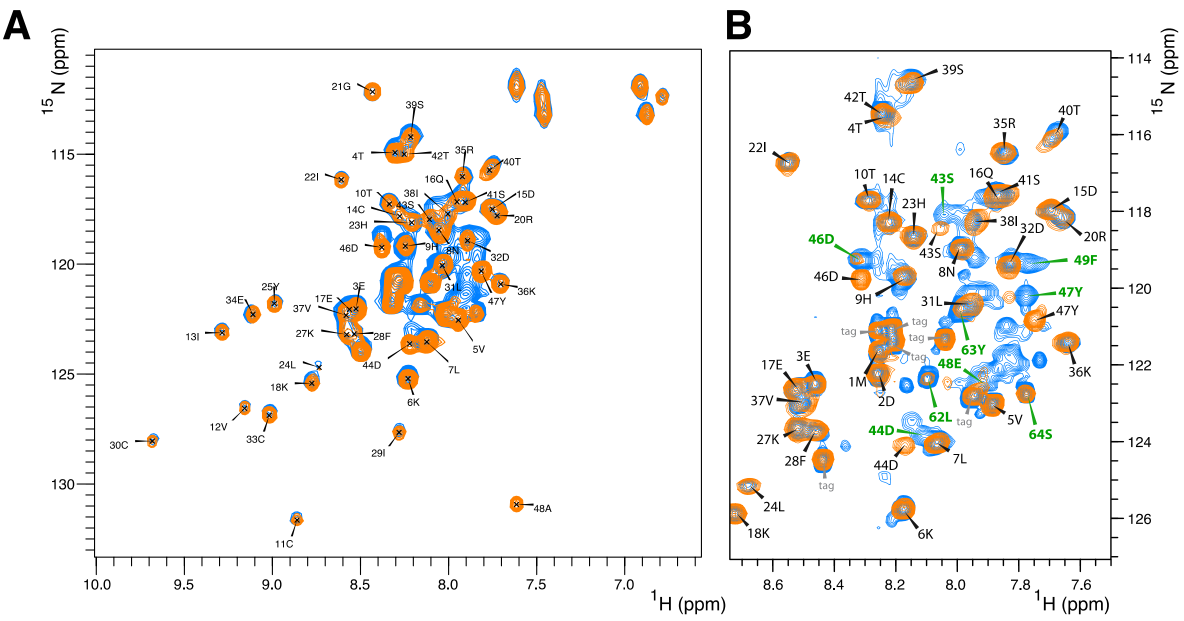

Figure S4 (Related to Figure 1): Wild type CsfB and CsfB^{A48E} observed by NMR spectroscopy. (A) ^{1}H-^{15}N SOFAST HMQC of CsfB (orange) overlaid onto CsfB^{A48E} (blue). Note the chemical shift conservation of the N-terminal folded domain and the appearance of new peaks at around 8ppm in proton dimension corresponding to the C-terminal region in CsfB^{A48E} spectrum (blue). **(B)** ^{1}H-^{15}N TROSY HSQC of CsfB^{WT} (orange) overlaid onto CsfB^{A48E} (blue) zoomed in on the central region of the spectra. Assigned peaks of CsfB are labelled in black. Extra or shifted peaks in the CsfB^{A48E} spectrum are labelled in green and peaks arising from the N-terminal His tag are labelled in grey.

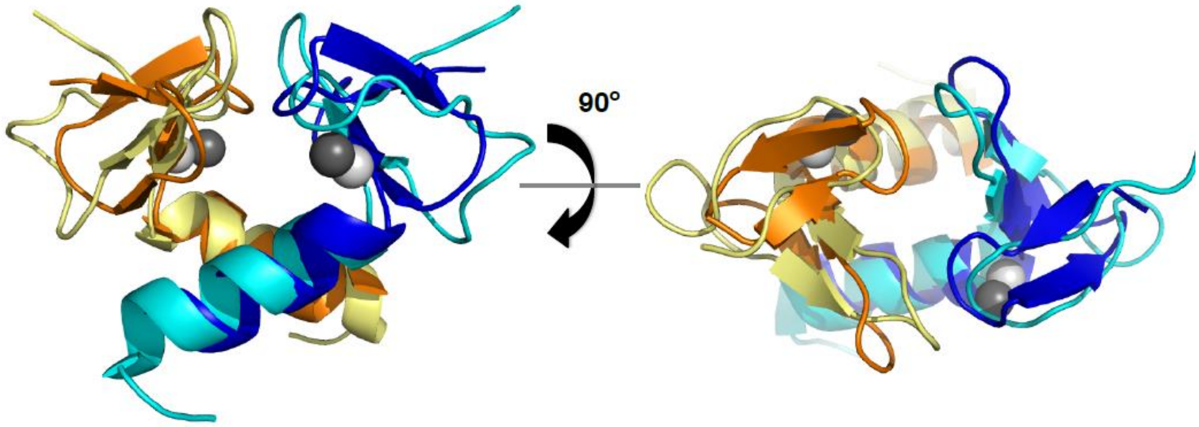

Figure S5 (Related to Figure 4): Structural alignment of CsfB and the ClpX N-terminal domain (NTD). The CsfB structure from this work (PDB: 5N7Y) is shown in orange and blue, and the ClpX NTD (PDB: 2DS6; (Park et al., 2007)) is shown in light yellow and cyan.

SUPPLEMENTAL TABLES S1-S5

Table S1 (Related to Figure 1). Protein engineering strategy to isolate a stabilised CsfB variant.

Mutation	Mutation Type	Expressed?	EI MS/NMR HSQC Results
A48P	Proline concept	Y	Stable full length protein
A48L	Conserved aliphatic	N	-
A48E	Charge principle	Y	Stable full length protein
F49A	Alanine substitution	Y	-
F49L	Conserved aliphatic	N	-
Y50F	Conserved bulk	Y	-
Y50E	Conserved charge	Y	-
K52A	Alanine substitution	Y	15% full length protein, 85% cleavage to residue 52
K53A	Alanine substitution	Y	-
K55A	Alanine substitution	Y	-

Two patches of conserved aromatic and basic residues near the C-terminus of CsfB, typical targets for trypsin-like and chymotrypsin-like proteases, were selected for mutation. Mutations were based on the proteolytic-halting charge principle and proline concept, as well as conservative mutations and classic alanine substitutions (Fellinger et al., 2008; Markert et al., 2003; Pace and Scholtz, 1998). Eight constructs were successfully expressed, but SDS and native PAGE gels from purification were inconclusive. Three constructs were taken forward to be assessed by electrospray ionization mass spectrometry (Figure S1) and NMR HSQC (Figure S4) for protein length and stability, two of which were found to be full-length and stable over time (A48P and A48E). CsfB^{A48E}, which was found to have comparable activity to wild-type CsfB *in vivo* (Figure S2), was used for future studies.

Table S2 (Related to Figure 3). Geometry of the zinc coordination centre of CsfB.

Averaged distance and angular values (for both monomers) for first and second coordination shell across the NMR ensemble.

	**Cys 11**	**Cys 14**	**Cys 30**	**Cys 33**
First coordination shell				
Sγ-Zn distance (Å)	2.30 ± 0.02	2.31 ± 0.01	2.29 ± 0.01	2.30 ± 0.01
Cβ-Sγ-Zn angle (º)	110.4 ± 4.5	111.6 ± 1.2	109.1 ± 1.1	110.7 ± 2.3
Second coordination shell				
Sγ-HN (i+2) distance (Å)	2.28 ± 0.11	4.57 ± 0.91	2.77 ± 0.23	6.39 ± 0.37
Sγ-H-N (i+2) angle (º)	145.5 ± 3.8	131.3 ± 6.7	151.1 ± 7.7	116.2 ± 2.3

Table S3 (Related to STAR Methods). Plasmids used in this study.

Plasmid	Description[a,b]	Source
***For expression in/purification from* E. coli**		
pET-46-CsfB	*6xHis-CsfB lacI amp*	This study
pNIC28-CsfB	*6xHis-TEV-CsfB lacI kan*	This study
pNIC28-CsfB^{A48P}	*6xHis-TEV-CsfB*^{A48P} *lacI kan*	This study
pNIC28-CsfB^{A48L}	*6xHis-TEV-CsfB*^{A48L} *lacI kan*	This study
pNIC28-CsfB^{A48E}	*6xHis-TEV-CsfB*^{A48E} *lacI kan*	This study
pNIC28-CsfB^{F49A}	*6xHis-TEV-CsfB*^{F49A} *lacI kan*	This study
pNIC28-CsfB^{F49L}	*6xHis-TEV-CsfB*^{F49L} *lacI kan*	This study
pNIC28-CsfB^{Y50F}	*6xHis-TEV-CsfB*^{Y50F} *lacI kan*	This study
pNIC28-CsfB^{Y50E}	*6xHis-TEV-CsfB*^{Y50E} *lacI kan*	This study
pNIC28-CsfB^{K52A}	*6xHis-TEV-CsfB*^{K52A} *lacI kan*	This study
pNIC28-CsfB^{K53A}	*6xHis-TEV-CsfB*^{K53A} *lacI kan*	This study
pNIC28-CsfB^{K55A}	*6xHis-TEV-CsfB*^{K55A} *lacI kan*	This study
pLATE31-CsfB	*CsfB-6xHis lacI amp*	This study
pET-46-SigG	*6xHis-sigG lacI amp*	This study
pET28-TxrA-SigE	*6xHis-Trx-TEV-sigE*$^{17\text{-}239}$ *lacI kan*	This study
***For expression in/modification of* B. subtilis**		
pJJ46	*amyE*::P_{spank}-*sigG lacI spc, amp*	This study
pSFO1	*amyE*::$P_{hyperspank}$-*sigE*$^{17\text{-}239}$ *lacI spc, amp*	This study
pAH88	*thrC*::$P_{hyperspank}$-*csfB lacI erm, amp*	This study
pKF70	*thrC*::$P_{hyperspank}$-*csfB*^{A48E} *lacI erm, amp*	This study
pKF87	*thrC*::$P_{hyperspank}$-*csfB*^{V37A} *lacI erm, amp*	This study
pKF88	*thrC*::$P_{hyperspank}$-*csfB*^{V37E} *lacI erm, amp*	This study
pKF89	*thrC*::$P_{hyperspank}$-*csfB*^{I38A} *lacI erm, amp*	This study
pKF90	*thrC*::$P_{hyperspank}$-*csfB*^{I38E} *lacI erm, amp*	This study
pKF91	*thrC*::$P_{hyperspank}$-*csfB*^{V37A,I38A} *lacI erm, amp*	This study
pTK2	*thrC*::$P_{hyperspank}$-*csfB*$^{1\text{-}48}$ *lacI erm, amp*	This study
pAH334	*sacA*::P_{spoIID}-*luxABCDE cat*, *amp*	This study
pAH336	*sacA*::P_{sspB}-*luxABCDE cat*, *amp*	This study

[a]Antibiotic resistance genes are referred to as follows: *amp* (ampicillin), *cat* (chloramphenicol), *erm* (erythromycin plus lincomycin), and *kan* (kanamycin).

[b]See *Plasmid construction* section of STAR Methods for details.

Table S4 (Related to STAR Methods). Oligonucleotides used in this study.

Oligonucleotide	Sequence (5’ → 3’)*
CsfB pET46 Fw	gacgacgacaagatggacgaaacagttaaac
CsfB pET46 Rv	gaggagaagcccggttatgaatataatggcggtg
CsfB pLATE31 Fw	agaaggagatataactatggacgaaacagtt
CsfB pLATE31 Rv	gtggtggtgatggtgatggcctgaatataatggcgg
CsfB *1-59* pNIC28 Fw	tacttccaatccatggacgaaacagttaaac
CsfB *1-59* pNIC28 Rv	tatccacctttactgtcatgtatgaatgctctttag
CsfB A48P mut Fw	catcaacttctgatcctgactatCcgttttacgtaaaaaaactaaagagcattc
CsfB A48P mut Rv	gaatgctctttagtttttttacgtaaaacgGatagtcaggatcagaagttgatg
CsfB A48L mut Fw	catcaacttctgatcctgactatTTgttttacgtaaaaaaactaaagagcattcatacac
CsfB A48L mut Rv	gtgtatgaatgctctttagtttttttacgtaaaacAAatagtcaggatcagaagttgatg
CsfB A48E mut Fw	cttctgatcctgactatgAgttttacgtaaaaaaactaaagagcattccatacacc
CsfB A48E mut Rv	ggtgtatggaatgctctttagtttttttacgtaaaacTcatagtcaggatcagaag
CsfB F49A mut Fw	caacttctgatcctgactatgcgGCttacgtaaaaaaactaaagagc
CsfB F49A mut Rv	gctctttagtttttttacgtaaGCcgcatagtcaggatcagaagttg
CsfB F49L mut Fw	catcaacttctgatcctgactatgcgCtttacgtaaaaaaactaaagagc
CsfB F49L mut Rv	gctctttagtttttttacgtaaaGcgcatagtcaggatcagaagttgatg
CsfB Y50F mut Fw	ctacatcaacttctgatcctgactatgcgttttTcgtaaaaaaactaaagagc
CsfB Y50F mut Rv	gctctttagtttttttacgAaaaacgcatagtcaggatcagaagttgatgtag
CsfB Y50E mut Fw	catcaacttctgatcctgactatgcgtttGaGgtaaaaaaactaaagagc
CsfB Y50E mut Rv	gctctttagtttttttacCtCaaacgcatagtcaggatcagaagttgatg
CsfB K52A mut Fw	cgttttacgtaGCaaaactaaagagcattcatacaccgcc
CsfB K52A mut Rv	ggcggtgtatgaatgctctttagttttGCtacgtaaaacg
CsfB K53A mut Fw	cgttttacgtaaaaGCactaaagagcattcatacaccgcc
CsfB K53A mut Rv	ggcggtgtatgaatgctctttagtGCttttacgtaaaacg
CsfB K55A mut Fw	cgttttacgtaaaaaaactaGCAagcattcatacaccgccattatattc
CsfB K55A mut Rv	gaatataatggcggtgtatgaatgcttGCtagtttttttacgtaaaacg
SigG pET46 Fw	gacgacgacaagatgtcgagaaataaagtcg
SigG pET46 Rv	gaggagaagcccggttattgatgaatatttttattc
SigE *17-239* pET28 Fw	cgcggatccatgaaactgggcctgaaaagtga
SigE *17-239* pET28 Rv	ttgcctcgagttacaccattttgttaaattctttgcgca
JJ30 (*sigG* Fw)	*gtgagcggataacaattaagcttagtcgac*gtacagcagctcctgtag
JJ31 (*sigG* Rv)	*ccgaattagcttgcatgcggctagc*ttattgatgaatatttttattcatttgtttgatag

continued →

**Oligonucleotide**	**Sequence (5' → 3')***
← *continued*	
AH41 (*csfB* Fw HindIII)	gatcaagctttacggaggtggagaagatg
AH42 (*csfB* Rv NheI)	gatcgctagctctactacgttcaatccttaaac
AH43 (Δ*sigE* P1)	aaatctatttagatgtcatttggctg
AH44 (Δ*sigE* P2)	*caattcgccctatagtgagtcgt*catcttcctctcccttctaaatg
AH45 (Δ*sigE* P3)	*ccagcttttgttccctttagtgag*taaaaaattttatggttagaaccccttg
AH46 (Δ*sigE* P4)	ccaaaacgtaaaccatccataatc
AH58 (P_{spoIID} Fw EcoRI)	gatcgaattcgatgagtctgcttctgagcaag
AH59 (P_{spoIID} Rv SalI)	gatcgtcgactgctcgggattcgactctag
AH60 (P_{sspB} Fw EcoRI)	gatcgaattcacgagatacatgaactgatgc
AH61 (P_{sspB} Rv SalI)	gatcgtcgactttttatttagtatggttgggttaactg
AH310 (MCS linker Fw)	aattcacagagctctcggcggccgcataactagtaagg
AH311 (MCS linker Rv)	tcgaccttactagttatgcggccgccgagagctctgtg
AH312 (BamHI mut Fw)	gcaatttctctgtcttaaagAatcctgaggaggaaaacagg
AH313 (BamHI mut Rv)	cctgttttcctcctcaggatTctttaagacagagaaattgc

*Restriction sites are underlined, regions matching plasmid sequences are italicized, and mutations are in uppercase

Table S5 (Related to STAR Methods). *B. subtilis* strains used in this study

Strain	Genotype[a,b,c]	Source
Strains for* in vivo *σ^G-functionality tests		
KF286	*ΔsigG*::*kan* Δ*csfB*::*tet* *sacA*::P_{sspB}-*luxABCDE cat*	This study
KF287	*ΔsigG*::*kan* Δ*csfB*::*tet* *sacA*::P_{sspB}-*luxABCDE cat* *amyE*::P_{spank}-*sigG lacI spc*	This study
KF288	*ΔsigG*::*kan* Δ*csfB*::*tet* *sacA*::P_{sspB}-*luxABCDE cat* *amyE*::P_{spank}-*sigG lacI spc* *thrC*::P_{spank}-*csfB lacI erm*	This study
KF386	*ΔsigG*::*kan* Δ*csfB*::*tet* *sacA*::P_{sspB}-*luxABCDE cat* *amyE*::P_{spank}-*sigG lacI spc* *thrC*::P_{spank}-*csfB*$^{1\text{-}48}$ *lacI erm*	This study
KF289	*ΔsigG*::*kan* Δ*csfB*::*tet* *sacA*::P_{sspB}-*luxABCDE cat* *amyE*::P_{spank}-*sigG lacI spc* *thrC*::P_{spank}-*csfB*^{A48E} *lacI erm*	This study
SFB9	*ΔsigG*::*kan* Δ*csfB*::*tet* *sacA*::P_{sspB}-*luxABCDE cat* *amyE*::P_{spank}-*sigG lacI spc* *thrC*::P_{spank}-*csfB*^{V37A} *lacI erm*	This study
SFB11	*ΔsigG*::*kan* Δ*csfB*::*tet* *sacA*::P_{sspB}-*luxABCDE cat* *amyE*::P_{spank}-*sigG lacI spc* *thrC*::P_{spank}-*csfB*^{V37E} *lacI erm*	This study
SFB15	*ΔsigG*::*kan* Δ*csfB*::*tet* *sacA*::P_{sspB}-*luxABCDE cat* *amyE*::P_{spank}-*sigG lacI spc* *thrC*::P_{spank}-*csfB*^{I38A} *lacI erm*	This study
SFB13	*ΔsigG*::*kan* Δ*csfB*::*tet* *sacA*::P_{sspB}-*luxABCDE cat* *amyE*::P_{spank}-*sigG lacI spc* *thrC*::P_{spank}-*csfB*^{I38E} *lacI erm*	This study
SFB17	*ΔsigG*::*kan* Δ*csfB*::*tet* *sacA*::P_{sspB}-*luxABCDE cat* *amyE*::P_{spank}-*sigG lacI spc* *thrC*::P_{spank}-*csfB*^{V37A,I38A} *lacI erm*	This study
Strains for* in vivo *σ^E-functionality tests		
SFB42	*ΔsigE*::*(erm)*::*phleo* Δ*csfB*::*tet* *sacA*::P_{spoIID}-*luxABCDE cat*	This study
SFB33	*ΔsigE*::*(erm)*::*phleo* Δ*csfB*::*tet* *sacA*::P_{spoIID}-*luxABCDE cat* *amyE*::$P_{hyperspank}$-*sigE*$^{17\text{-}end}$ *lacI spc*	This study
SFB38	*ΔsigE*::*(erm)*::*phleo* Δ*csfB*::*tet* *sacA*::P_{spoIID}-*luxABCDE cat* *amyE*::$P_{hyperspank}$-*sigE*$^{17\text{-}end}$ *lacI spc* *thrC*::P_{spank}-*csfB lacI erm*	This study
KCB32	*ΔsigE*::*(erm)*::*phleo* Δ*csfB*::*tet* *sacA*::P_{spoIID}-*luxABCDE cat* *amyE*::$P_{hyperspank}$-*sigE*$^{17\text{-}end}$ *lacI spc* *thrC*::P_{spank}-*csfB*$^{1\text{-}48}$ *lacI erm*	This study
SFB44	*ΔsigE*::*(erm)*::*phleo* Δ*csfB*::*tet* *sacA*::P_{spoIID}-*luxABCDE cat* *amyE*::$P_{hyperspank}$-*sigE*$^{17\text{-}end}$ *lacI spc* *thrC*::P_{spank}-*csfB*^{A48E} *lacI erm*	This study
SFB52	*ΔsigE*::*(erm)*::*phleo* Δ*csfB*::*tet* *sacA*::P_{spoIID}-*luxABCDE cat* *amyE*::$P_{hyperspank}$-*sigE*$^{17\text{-}end}$ *lacI spc* *thrC*::P_{spank}-*csfB*^{V37A} *lacI erm*	This study
SFB50	*ΔsigE*::*(erm)*::*phleo* Δ*csfB*::*tet* *sacA*::P_{spoIID}-*luxABCDE cat* *amyE*::$P_{hyperspank}$-*sigE*$^{17\text{-}end}$ *lacI spc* *thrC*::P_{spank}-*csfB*^{V37E} *lacI erm*	This study

continued →

Strain	Genotype[a,b,c]	Source
← *continued*		
SFB54	*ΔsigE*::*(erm)*::*phleo* Δ*csfB*::*tet* *sacA*::P_{spoIID}-*luxABCDE cat* *amyE*::$P_{hyperspank}$-*sigE*$^{17\text{-}end}$ *lacI spc* *thrC*::P_{spank}-*csfB*^{I38A} *lacI erm*	This study
SFB56	*ΔsigE*::*(erm)*::*phleo* Δ*csfB*::*tet* *sacA*::P_{spoIID}-*luxABCDE cat* *amyE*::$P_{hyperspank}$-*sigE*$^{17\text{-}end}$ *lacI spc* *thrC*::P_{spank}-*csfB*^{I38E} *lacI erm*	This study
SFB58	*ΔsigE*::*(erm)*::*phleo* Δ*csfB*::*tet* *sacA*::P_{spoIID}-*luxABCDE cat* *amyE*::$P_{hyperspank}$-*sigE*$^{17\text{-}end}$ *lacI spc* *thrC*::P_{spank}-*csfB*^{V37A,I38A} *lacI erm*	This study

[a]All strains are isogenic with the prototrophic wild type strain PY79 (Youngman et al., 1984)

[b]Antibiotic resistance genes are referred to as follows: *cat* (chloramphenicol), *erm* (erythromycin plus lincomycin), *kan* (kanamycin), *phleo* (phleomycin), *spc* (spectinomycin), and *tet* (tetracycline).

[c]For information on the sources of gene deletions, reporter genes, and other constructs, see *Plasmid construction* and *Strain construction* sections of the STAR Methods for details.
