# Supplementary material for: Structural and Functional Insights into Bacillus subtilis Sigma Factor Inhibitor, CsfB
Source: Structure. 2018 Apr 3;26(4):640–648.e5. doi: 10.1016/j.str.2018.02.007 (PMC5890618; doi:10.1016/j.str.2018.02.007)
Supplement: Document S2. Article plus Supplemental Information [file mmc2.pdf]

Short Article

Structure

Structural and Functional Insights into *Bacillus subtilis* Sigma Factor Inhibitor, CsfB

Graphical Abstract

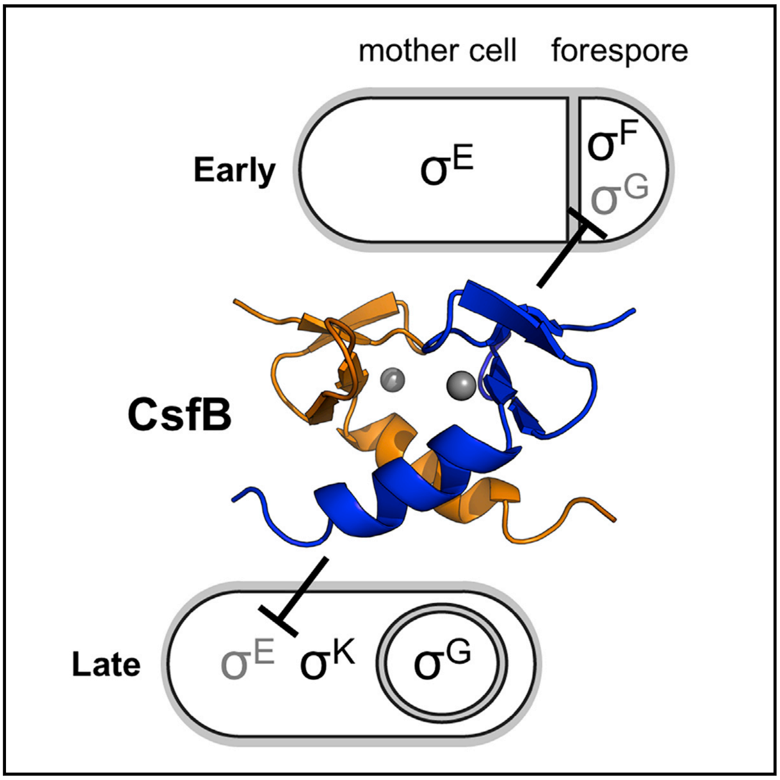

Authors

Santiago Martínez-Lumbreras, Caterina Alfano, Nicola J. Evans, ..., Sarah Fixon-Owoo, Amy H. Camp, Rivka L. Isaacson

Correspondence

rivka.isaacson@kcl.ac.uk

In Brief

Martínez-Lumbreras, Alfano et al. have solved the structure of the anti-sigma factor CsfB and explored its role in inhibiting two alternative sigma factors during *Bacillus subtilis* spore formation. The results provide insight into the molecular mechanism underlying a gene expression switch in bacteria.

Highlights

- The structure of CsfB is unique among anti-sigma factors
- CsfB assembles into a tight homodimer of treble-clef zinc finger domains
- CsfB dimerization is essential for inhibition of two alternative sigma factors

Martínez-Lumbreras et al., 2018, Structure *26*, 640–648
April 3, 2018 © 2018 The Authors. Published by Elsevier Ltd.
https://doi.org/10.1016/j.str.2018.02.007

CellPress

Structure
Short Article

Structural and Functional Insights into *Bacillus subtilis* Sigma Factor Inhibitor, CsfB

Santiago Martínez-Lumbreras,[1,5] Caterina Alfano,[1,2,5] Nicola J. Evans,[1] Katherine M. Collins,[1] Kelly A. Flanagan,[4] R. Andrew Atkinson,[3] Ewelina M. Krysztofinska,[1] Anupama Vydyanath,[1] Jacquelin Jackter,[4] Sarah Fixon-Owoo,[4] Amy H. Camp,[4] and Rivka L. Isaacson[1,6,*]
[1]Department of Chemistry, King's College London, Britannia House, 7 Trinity Street, London SE1 1DB, UK
[2]Structural Biology and Biophysics Unit, Fondazione Ri.MED, Via Bandiera, 11, 90133 Palermo, Italy
[3]Centre for Biomolecular Spectroscopy and Randall Division of Cell and Molecular Biophysics, King's College London, New Hunt's House, Guy's Campus, London SE1 1UL, UK
[4]Department of Biological Sciences, Mount Holyoke College, 50 College Street, South Hadley, MA 01075, USA
[5]These authors contributed equally
[6]Lead Contact
*Correspondence: rivka.isaacson@kcl.ac.uk
https://doi.org/10.1016/j.str.2018.02.007

SUMMARY

Global changes in bacterial gene expression can be orchestrated by the coordinated activation/deactivation of alternative sigma (σ) factor subunits of RNA polymerase. Sigma factors themselves are regulated in myriad ways, including via anti-sigma factors. Here, we have determined the solution structure of anti-sigma factor CsfB, responsible for inhibition of two alternative sigma factors, σ^G and σ^E, during spore formation by *Bacillus subtilis*. CsfB assembles into a symmetrical homodimer, with each monomer bound to a single Zn^{2+} ion via a treble-clef zinc finger fold. Directed mutagenesis indicates that dimer formation is critical for CsfB-mediated inhibition of both σ^G and σ^E, and we have characterized these interactions *in vitro*. This work represents an advance in our understanding of how CsfB mediates inhibition of two alternative sigma factors to drive developmental gene expression in a bacterium.

INTRODUCTION

Eukaryotic and prokaryotic cells alike possess the ability to alter their phenotypes through global changes in gene expression. In bacteria, these transitions enable survival during stress conditions, drive developmental programs, and promote infection of host organisms. One common mechanism bacteria utilize to effect large-scale changes in gene expression is through alternative sigma (σ) factor subunits of RNA polymerase (RNAP). The dissociable RNAP sigma factor subunit is responsible for recognition of promoter DNA and the subsequent initiation of transcription. Most sigma factors are members of the σ^{70} superfamily, which is subdivided into four classes based upon conservation and the presence/absence of the conserved sigma domains (σ1.1, σ2, σ3, and σ4) that mediate interactions with RNAP and/or promoter DNA (reviewed in Feklistov et al., 2014; Paget, 2015). All bacteria employ an essential primary sigma factor (class I) that directs transcription of housekeeping genes; many bacteria also possess alternative sigma factors (classes II, III, and IV) that compete for binding to RNAP and redirect it to transcribe sets of genes required for adaptive responses. Hence, the suite of genes expressed in a bacterial cell can be reprogrammed by manipulating the levels, activity, or availability of alternative sigma factors (reviewed in Osterberg et al., 2011).

One prevalent form of post-translational regulation of alternative sigma factors occurs via anti-sigma factors: proteins that bind to and prevent their cognate sigma factor from interacting with RNAP. Unlike sigma factors, which share sequence, structural, and functional conservation, anti-sigma factors are more diverse in their sequences, structures, and/or mode of sigma factor inhibition (reviewed in Paget, 2015). A number of structural and bioinformatics analyses have revealed that anti-sigma factors for the class IV extracytoplasmic function (ECF) sigma factors often share one of two conserved anti-sigma domain structures, despite little sequence conservation (reviewed in Campagne et al., 2015). Less is known, however, of the structural features of anti-sigma factors that antagonize non-class IV alternative sigma factors, given the limited number of structures determined to date (Campbell et al., 2002; Masuda et al., 2004; Sorenson et al., 2004).

Here, we have structurally analyzed CsfB (also called Gin), a small, Zn^{2+}-binding anti-sigma factor that inhibits two class III alternative sigma factors during spore formation by the model bacterium *Bacillus subtilis* (Figure 1A) (Chary et al., 2007; Decatur and Losick, 1996; Karmazyn-Campelli et al., 2008; Rhayat et al., 2009; Serrano et al., 2011, 2015). In the forespore cell (the nascent spore), CsfB binds and inhibits the late-acting sigma factor σ^G, helping to ensure that it does not become active before the early-acting sigma factor σ^F has completed its program of gene expression (Karmazyn-Campelli et al., 2008; Rhayat et al., 2009). In the mother cell, which helps support the development of the forespore, CsfB binds the early-acting sigma

 © 2018 The Authors. Published by Elsevier Ltd.
This is an open access article under the CC BY license (http://creativecommons.org/licenses/by/4.0/).

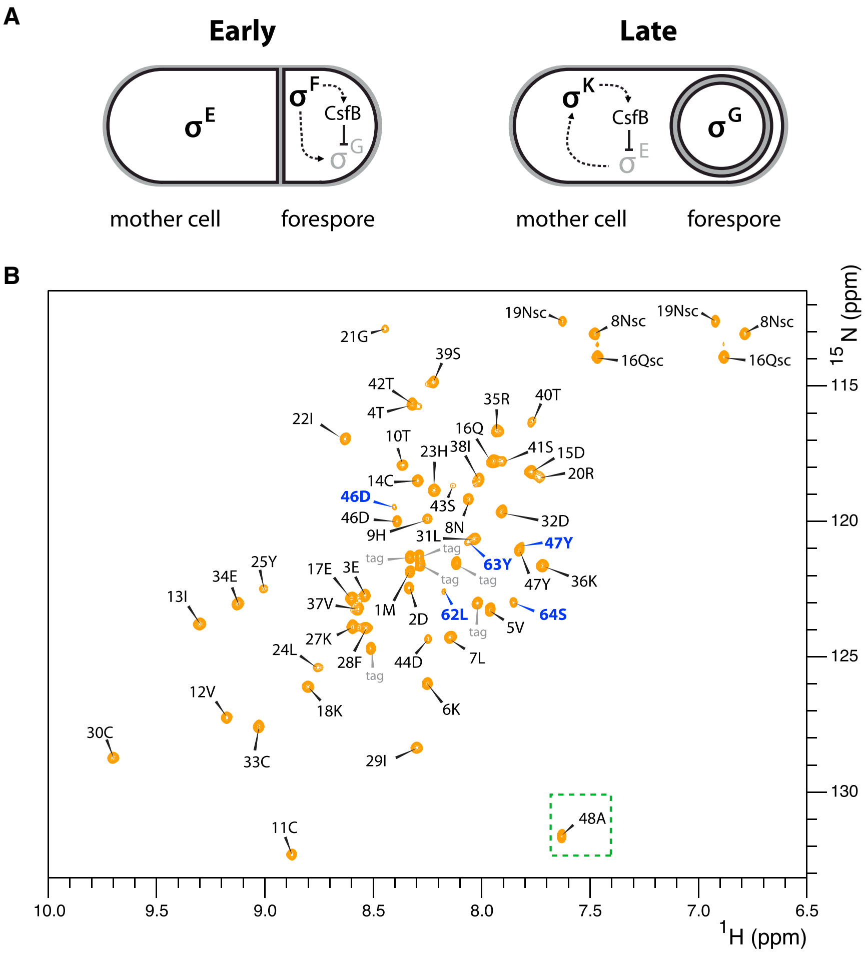

Figure 1. The Anti-sigma Factor CsfB Helps to Orchestrate the Switch from Early to Late Gene Expression during *B. subtilis* Sporulation

(A) Cartoon depiction of the role of the dual-specificity anti-sigma factor CsfB in regulating the transition from early to late gene expression during *B. subtilis* sporulation. Early in sporulation (reviewed in Tan and Ramamurthi, 2014), an asymmetric cell division event produces two cells: a smaller forespore (the nascent spore) and a larger mother cell. Initially, these two cells lie side-by-side; the mother cell then engulfs the forespore in a phagocytic-like process. At early times, σ^F and σ^E drive gene expression in the forespore and mother cell, respectively. Among the genes activated by σ^F and σ^E are those encoding the late-acting sigma factors, σ^G and σ^K, respectively (dashed arrows). The anti-sigma factor CsfB is expressed in both compartments under the control of σ^F and σ^K (dashed arrows). In the forespore, CsfB antagonizes σ^G at early times (barred line). In the mother cell, CsfB antagonizes σ^E at later times (barred line).

(B) ^{1}H-^{15}N HSQC spectrum of CsfB (orange). Full assignment of the cleaved CsfB version appears in black (CsfB$^{1-48}$), partial assignment of the residual full-length CsfB in blue and the tag residues in gray; sc denotes side chain resonances. The C-terminal residue from the cleaved version (A48) is highlighted by a green square.

factor σ^E, helping to inactivate it after the switch to σ^K (Serrano et al., 2015). Here, we report the structure of CsfB and characterize its interaction with σ^G and σ^E.

RESULTS

Recombinant CsfB Degrades to a Stable but Nonfunctional Domain

We produced recombinant N-terminally histidine-tagged full-length CsfB (residues 1–64), but the protein consistently degraded to a stable product comprising residues 1–48. The gradual disappearance of the C-terminal 16-amino acid fragment was confirmed by electrospray ionization mass spectrometry (Figure S1) and nuclear magnetic resonance (NMR) backbone assignment indicated that the predominant C-terminal residue was A48 (Figure 1B). We predicted that this shorter form of CsfB (CsfB$^{1-48}$) was nonfunctional, given the absence of residues required for σ^G inhibition (Rhayat et al., 2009). To confirm this, we assessed the ability of CsfB$^{1-48}$ to inhibit σ^G or σ^E when the proteins were co-expressed during vegetative growth of *B. subtilis*, an approach that has been used previously (Karmazyn-Campelli et al., 2008; Rhayat et al., 2009). Whereas wild-type CsfB inhibited >99% of σ^G activity and ~77% of σ^E activity, the CsfB$^{1-48}$ variant displayed no inhibition of either sigma factor (Figure S2).

Isolation of a Functional, Full-Length CsfB Protein *In Vitro*

Since CsfB$^{1-48}$ was unable to inhibit σ^G and σ^E, we adopted several approaches to obtain a full-length, stable version of CsfB. Initially, we produced a C-terminally histidine-tagged version of CsfB, which was slower to degrade but still consistently converted to the CsfB$^{1-48}$ species (Figure S3). We next rationally designed a panel of CsfB variants (Table S1) to identify a functional version of CsfB that remained full-length. Of these, A48E (altered at the known cleavage point) proved the most successful, yielding a stable full-length version of CsfB that remained intact for 4 days as confirmed by mass spectrometry (Figure S1). CsfB^{A48E} inhibited both σ^G and σ^E to the same extent as wild-type CsfB *in vivo* (Figure S2), suggesting that the A48E substitution does not alter protein function. Satisfyingly, the NMR HSQC spectrum of CsfB^{A48E} overlaid precisely with that of CsfB$^{1-48}$ (truncated wild-type), except for the presence of peaks corresponding to the additional C-terminal residues (Figure S4). Some of these additional peaks could be assigned from triple-resonance experiments and, upon revisiting earlier HSQC spectra of freshly purified wild-type CsfB, a low population of these same peaks was visible from the residual full-length protein that had not yet degraded (Figure 1B). Several peaks within the C-terminal region could not be assigned due to a line-broadening effect (Figure S4B). The new C-terminal peaks, whether assignable or not, displayed little dispersion in the proton dimension, a hallmark of low structural complexity.

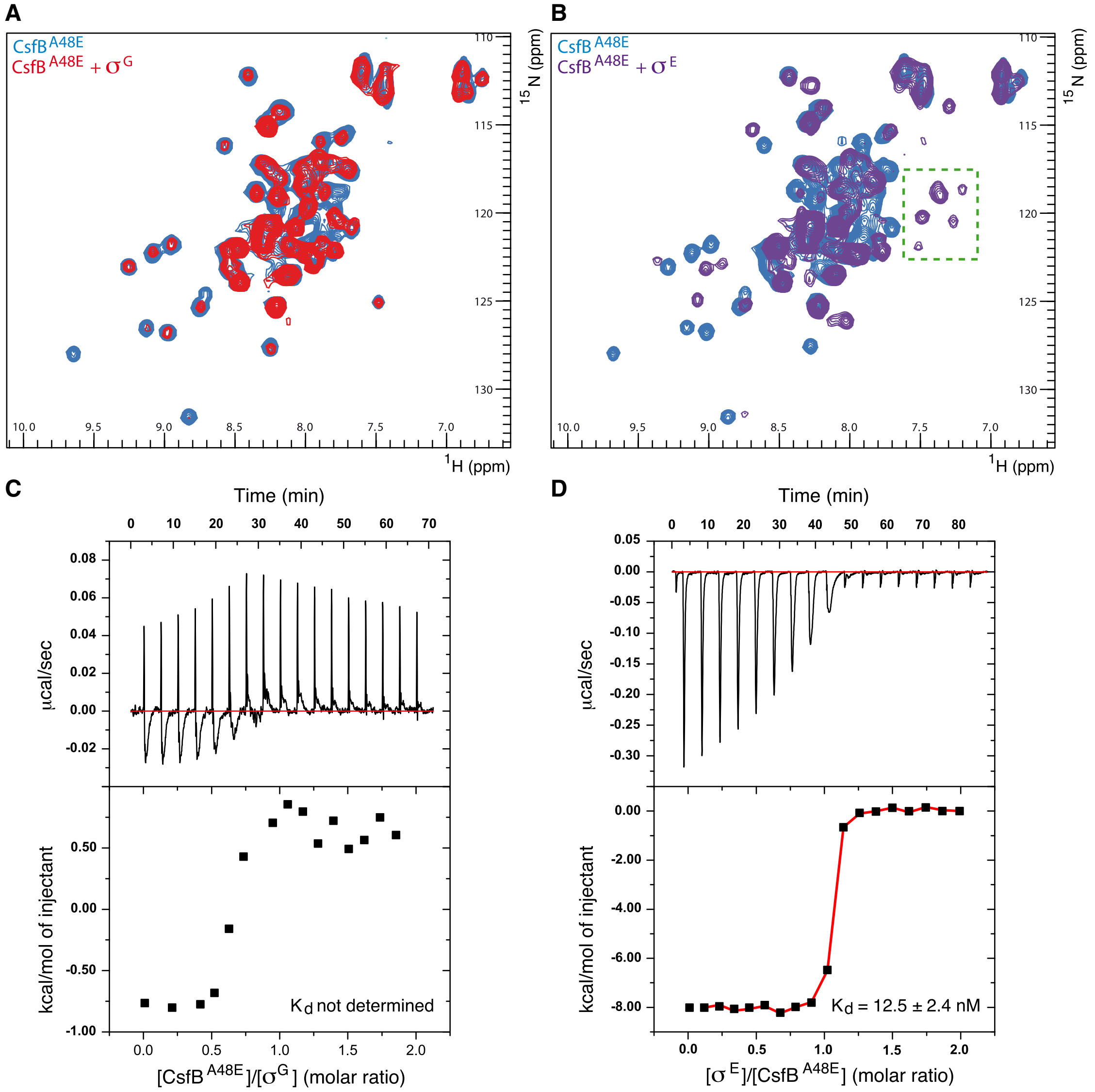

Figure 2. Interactions of CsfB^{A48E} with σ^G and σ^E

(A and B) Overlay of ^{1}H-^{15}N SOFAST HMQC spectra of ^{15}N-labeled CsfB^{A48E} alone (blue), and in presence of 2-fold molar excess of (A) σ^G (red) or (B) σ^E (purple). Extra peaks appearing upon titration with σ^E are highlighted by a green square.

(C and D) ITC thermograms of interaction between CsfB^{A48E} and (C) σ^G or (D) σ^E. Raw data (upper panels), binding isotherm (lower panels). Fitted data for CsfB^{A48E}-σ^E interaction: $\Delta H = -8.04 \pm 0.04$ kcal/mol; $\Delta S = 9.19 \pm 0.50$ cal/(mol·K); N = 1.01 ± 0.00 sites.

Interaction of CsfB^{A48E} with σ^G and σ^E

With the functional, full-length CsfB^{A48E} protein in hand, we first analyzed its interactions with its target sigma factors. To this end, we produced recombinant full-length σ^G (residues 1–260) and a truncated version of σ^E (residues 17–239) lacking the N-terminal membrane-anchored pro-sequence (Peters et al., 1992). We then carried out NMR chemical shift perturbation (CSP) analysis between unlabeled σ^G or σ^E and ^{15}N-labeled CsfB^{A48E}. Titration of unlabeled σ^G caused the majority of CsfB^{A48E} backbone amide signals to gradually disappear (Figure 2A). This result indicates an interaction between CsfB^{A48E} and σ^G, although the disappearance of most peaks prevented identification of specific positions on CsfB^{A48E} that mediate contact. As a control, we performed CSP analysis between unlabeled σ^G and ^{15}N-labeled CsfB$^{1-48}$, the truncated variant incapable of inhibiting σ^G *in vivo*. Consistent with the inability of these proteins to interact, no changes to the CsfB$^{1-48}$ backbone amide signals were observed.

When ^{15}N-labeled CsfB^{A48E} was titrated with unlabeled σ^E, many CsfB^{A48E} backbone amide signals decreased in intensity and shifted position significantly (Figure 2B), indicating a tight interaction in the nanomolar to low micromolar affinity range. As a result of the slow timescale, it was not possible to reliably assign the peaks in their new positions and, unfortunately, the resulting complex was too large for the triple-resonance experiments required to assign the bound state. Hence, we could not assess the relative contributions of each of the bound residues. However, we noted that addition of σ^E caused several of the

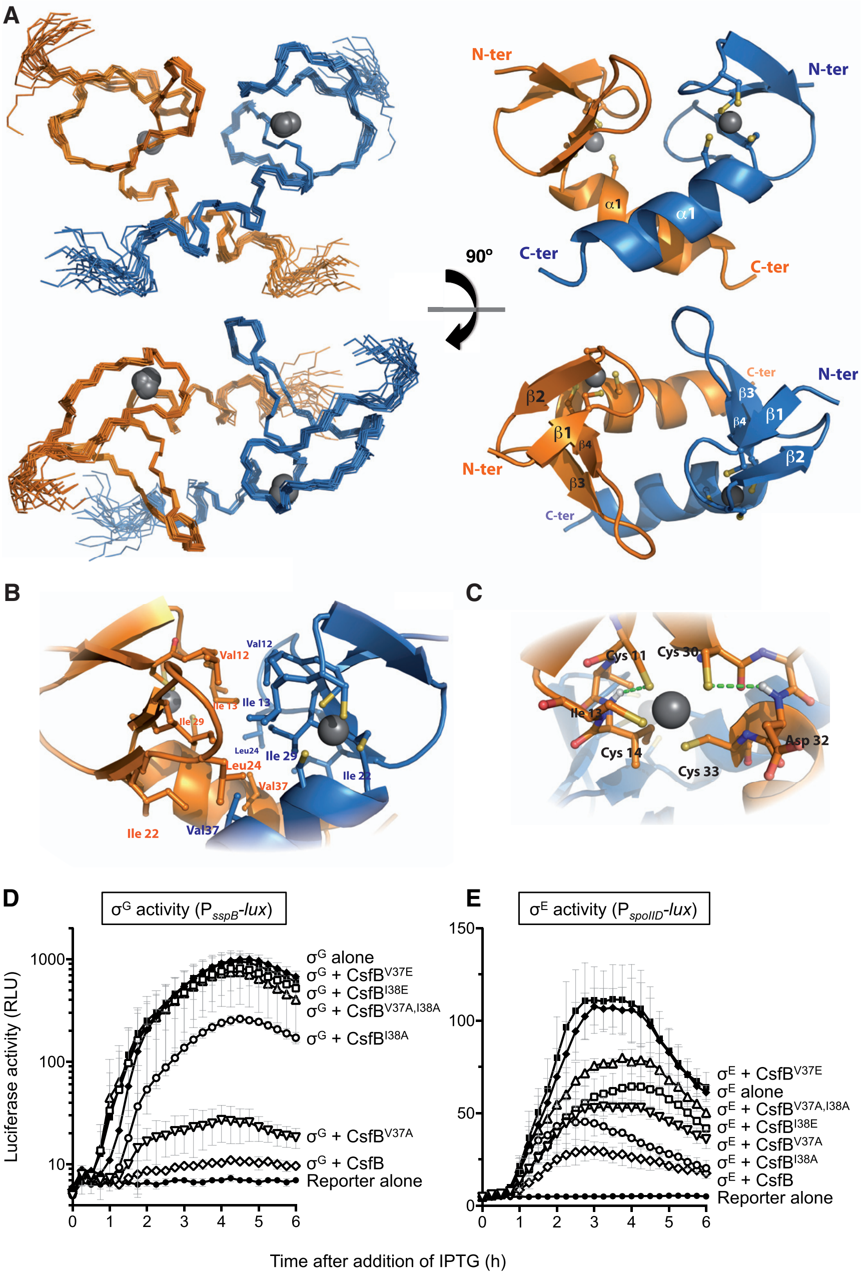

Figure 3. NMR Solution Structure of the CsfB$^{1–48}$ Dimer and Functionality of Dimerization-Deficient CsfB Variants

(A) Orthogonal views of ensemble backbone (left) and cartoon (right) representations for the 20 lowest energy ARIA-calculated structures as deposited in the PDB (PDB: 5N7Y).

(B) Detailed view of the dimer interface; hydrophobic buried residues are depicted using ball and stick representation.

(C) Detailed view of the zinc finger coordination shell showing the cysteine residues and the Sγ(i)-HN(i+2) hydrogen bonds (green dashed lines) in the first and second spheres of coordination.

(D and E) CsfB variants lacking putative dimerization residues V37 and/or I38 are deficient for sigma factor inhibition *in vivo*. Vegetatively growing *B. subtilis* cells were induced with IPTG to express (D) σ^G or (E) σ^E alone or in combination with wild-type or variant CsfB. Sigma factor activity was monitored by light production (measured in relative light units [RLU]) from σ^G- or σ^E-dependent luciferase reporter genes (P_{sspB}-*lux* or P_{spoIID}-*lux*, respectively). Control strains lacking inducible constructs ("Reporter alone") are shown for comparison in each graph. Error bars indicate SD. Strains used in this assay are listed in Table S5.

peaks corresponding to CsfB^{A48E} residues 49–64 to shift to the ^{1}H upfield region of the spectrum (Figure 2B), suggesting that the C-terminal region becomes more structured upon interaction with σ^E. As expected, σ^E caused no shifts in the spectrum of the ^{15}N-labeled CsfB$^{1-48}$ truncated variant.

We next carried out isothermal titration calorimetry (ITC) to quantify the interaction between CsfB^{A48E} and its two cognate sigma factors. The CsfB^{A48E}-σ^E interaction was determined to have a K_d of 12.5 ± 2.4 nM (Figure 2D) and 1:1 stoichiometry. CsfB^{A48E} and σ^G also showed clear evidence of an interaction (Figure 2C), although a K_d and stoichiometry could not be determined, possibly due to instability or aggregation of our recombinant σ^G. However, by comparing the ITC data to σ^E experiments, we can conclude that the CsfB^{A48E}-σ^G binding affinity is likely within the same order of magnitude. As a control, we verified that no interaction was observed between the truncated variant CsfB$^{1-48}$ and σ^G or σ^E under the same conditions.

NMR Solution Structure of CsfB

Next, we sought to solve the solution structure of CsfB. Despite having isolated a functional, full-length CsfB variant (CsfB^{A48E}), we could only obtain high-quality NMR triple-resonance signals for residues comprising the originally purified, truncated CsfB$^{1-48}$ variant. Given that the C-terminal 16 residues presented low structural complexity, and the folding of the rest of the protein was conserved, we opted to complete the full NMR assignments (BMRB: 34102) and solve the solution structure for CsfB residues 1–48. It forms a tight symmetrical homodimer (Figures 3A and 3B; PDB: 5N7Y; structural statistics in Table 1), where each monomer consists of a treble-clef zinc finger motif (Grishin, 2001).

The folded domain (residues 8–40) of each CsfB monomer contains two β hairpins separated by a short turn, followed by a C-terminal α helix. The structure clearly indicates that each monomer binds a Zn^{2+} ion; we confirmed a 1:1 Zn^{2+}:CsfB ratio by ICP-MS. Zinc binding by CsfB involves the coordination of two cysteine residues from the first β-hairpin knuckle (C11 and C14) and two additional cysteines from the first turn

Table 1. NMR and Refinement Statistics for the Final 20 Ensemble Structures of CsfB

NMR Distance and Dihedral Constraints (per Monomer)	
Distance constraints	
Total unambiguous constraints	1,154
Intra-residue	417
Sequential (|i-j| = 1)	231
Medium-range (1 < |i-j| < 4)	118
Long-range (|i-j| > 5)	201
Intermolecular	187
Ambiguous constraints	116
TALOS-derived dihedral constraints	
Total dihedral constraints (Φ+Ψ)	64
**Structure Statistics**	
Violations per structure (mean and SD)	
Number of violated distance restraints (>0.25 Å)	0.65 ± 0.63
Max. distance constraint violation	0.28 Å
Number of violated dihedral angle restraints (>5°)	2.3 ± 0.9
Max. dihedral angle violation	9.2°
Ramachandran plot analysis[a]	Residues 8–40
Residues in most favored regions	89.5% ± 4.0%
Residues in additionally allowed regions	10.5% ± 4.0%
Residues in disallowed regions	0.0% ± 0.0%
Derivation from idealized geometry	
Bond length (Å)	0.0079 ± 0.0005
Bond angles (°)	0.83 ± 0.05
Average RMSD to mean structure (range 8–40)	
Backbone	0.5 ± 0.2
Heavy	1.1 ± 0.2

[a]Obtained from PDB NMR structure validation report.

of the α helix (C30 and C33) in a tetrahedral conformation (Figure 3C), a classic treble-clef zinc finger fold (Krishna et al., 2003; Kaur and Subramanian, 2016). The chemical shift values for ^{13}Cα (~59 ppm) and ^{13}Cβ (~31 ppm) are consistent with zinc-binding character (Kornhaber et al., 2006). The second coordination shell is defined by the formation of two hydrogen bonds between the cysteine sulfur atoms (C11 and C30) and the amide group of the residue at position +2 (I13 and D32, respectively). For all zinc coordination parameters, see Table S2.

The CsfB homodimer interface spans 1,138 Å^2 (calculated by PISA; Krissinel and Henrick, 2007) and involves numerous intermolecular contacts between the β hairpins and α helices of each monomer (Figure 3B). Several nonpolar residues (V12, I13, I22, L24, I26, V37, I38) are embedded in the dimer interface, creating a hydrophobic core resembling that of a globular protein. In contrast, the surface of the protein displays hydrophilic side chains that create an intricate network of polar contacts. For example, the ε-amino group of K36 from one monomer is surrounded by the carboxylate side chain of D32 from the same chain and the hydroxyl group of Y25 from the other monomer. In addition, the side chains of K27 from one monomer and D15 from the other chain, as well as those from S41 and E34, form clear polar contacts.

Finally, we tested the effect of disrupting CsfB dimer formation in *B. subtilis* by constructing CsfB variants with substitutions at V37 and/or I38. These two residues in the α helix of one CsfB monomer pack against the same two residues in the α helix of the second monomer; these two positions are almost always occupied by hydrophobic residues in CsfB homologs (Rhayat et al., 2009; Camp et al., 2011). We found that the individual alanine substitutions (V37A and I38A) had only modest effects on CsfB-mediated inhibition of σ^G or σ^E in the vegetative co-induction assay (Figures 3D and 3E). In contrast, substitution of these residues with glutamate (V37E and I38E) significantly reduced inhibition of σ^G from nearly 100% to 0% and 15%, respectively. CsfB-mediated inhibition of σ^E was also significantly compromised by the V37E substitution (reduced from 77% to 9%), while the I38E had a more modest effect on inhibition (reducing it from 77% to 49%; Figures 3D and 3E). Lastly, we found that simultaneous substitution of these positions for alanine (V37A, I38A), also significantly diminished CsfB-dependent σ^G and σ^E inhibition, to only 17% and 32%, respectively. These findings imply that dimer formation by CsfB is required for inhibition of σ^G and σ^E.

DISCUSSION

Here, we have solved the solution structure of the folded domain of the anti-sigma factor CsfB, which inhibits two class III sigma factors, σ^G and σ^E, during *B. subtilis* sporulation. The two conserved C-X-X-C motifs of CsfB suggested early on that it was likely to bind Zn^{2+} (Karmazyn-Campelli et al., 2008), a prediction that was verified biochemically in two studies, albeit with different Zn^{2+}:CsfB ratios reported (Rhayat et al., 2009; Serrano et al., 2011). Genetic analyses further hinted that CsfB might function as a dimer (Rhayat et al., 2009). Our solved structure verifies that CsfB is a symmetric homodimer, with each monomer adopting a treble-clef zinc finger fold coordinating a single Zn^{2+} ion. We confirmed a 1:1 Zn^{2+}:CsfB ratio by ICP-MS, in line not only with our structure but also with the 1:1 Zn^{2+}:CsfB ratio reported by Serrano et al. (2011). Our CsfB structure also offers an explanation for the finding by Rhayat et al. (2009) that mutating the highly conserved glycine at position 21 to cysteine abolished CsfB function. The two alpha protons in this glycine point snugly into the hydrophobic core of the protein such that any other side chain at this position would likely cause a steric hindrance.

Our CsfB structure is inconsistent, however, with a model proposed by Rhayat et al. (2009) in which CsfB forms an asymmetric dimer that coordinates a single Zn^{2+} ion between different cysteine pairs on alternative monomers. This model was a sensible interpretation of data from a series of cysteine deletion mutants co-expressed *in vivo*, as well as their measurement of a 0.5:1 Zn^{2+}:CsfB ratio. Given that our NMR data clearly show the presence of a symmetric dimer (one subset of signals), we suspect that the Zn^{2+}:CsfB ratio reported by these authors may be an artifact of their maltose-binding protein-CsfB fusion, the functionality of which was

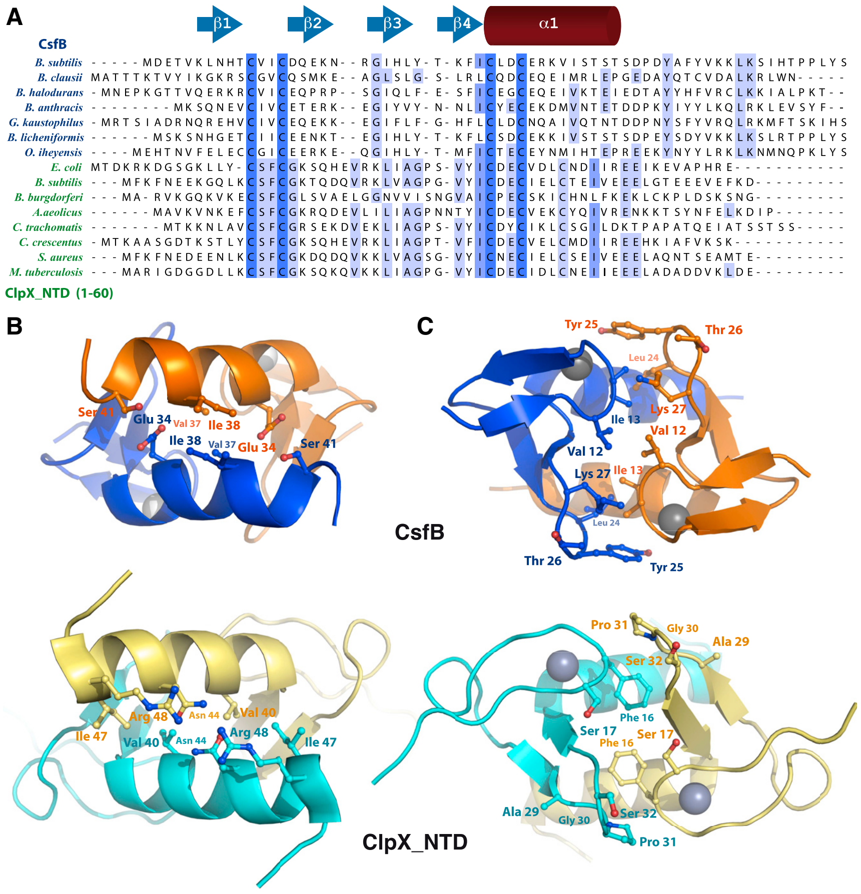

Figure 4. Structural and Sequence Comparison of CsfB with ClpX_NTD

(A) Sequence alignment of CsfB and ClpX_NTD from different species. Cartoons above the sequences represent the positions involved in secondary structure formation in CsfB.

(B and C) Structural comparison of dimer interfaces in CsfB (orange and blue, top) and ClpX_NTD (PDB: 2DS6, light yellow and cyan, bottom). Zinc cations shown as gray spheres. Residues involved in (B) antiparallel helices packaging and (C) loop contacts are shown for each.

not reported. Alternatively, it may be that purification of CsfB from sporulating *B. subtilis* accounts for the altered zinc content in the Rhayat et al. (2009) study; our CsfB protein and that of Serrano et al. (2011) were purified from *Escherichia coli*. In this case, it would be tempting to speculate that CsfB is subject to Zn^{2+}-dependent regulation during sporulation of *B. subtilis*, as has been suggested previously (Karmazyn-Campelli et al., 2008).

The CsfB and ClpX N-Terminal Domain Treble-Clef Dimers Are Structurally Similar

We have found here that CsfB adopts a treble-clef zinc finger fold, one of the most common zinc finger arrangements (Kaur and Subramanian, 2016; Krishna et al., 2003). Interestingly, CsfB appears most similar to the N-terminal domain (NTD) of the *E. coli* AAA + ATPase ClpX (PDB: 2DS5–2DS8) (Park et al., 2007); to our knowledge, CsfB and the ClpX NTD are the only examples of dimerization found in the treble-clef zinc finger fold family (as identified by the Dali server [Holm and Laakso, 2016; Holm and Rosenstrom, 2010]). ClpX is the chaperone/unfoldase component of the ClpXP protease, a barrel-shaped proteolytic machine that degrades target proteins for quality control as well as regulation (Baker and Sauer, 2012). It is proposed that the dimerized ClpX NTDs interact with substrates or cofactors of ClpXP and guide them toward the protease complex for degradation (Thibault et al., 2006).

CsfB and the ClpX NTD (PDB: 2DS6) structurally align with a root-mean-square deviation (RMSD) of 4.4 Å (backbone alignment between CsfB 8–43 and CplX 11–49 residues over 196 atoms) and share a sequence identity of ∼20% (Figures 4A and S5). Both homodimerize via a hydrophobic core of residues derived from their C-terminal α helices and two β hairpins. The proteins have a similar area of dimer interface at 945 $Å^2$ for the ClpX NTD (Donaldson et al., 2003) and 1,138 $Å^2$ for CsfB. Although both dimers are held together with a combination of hydrophobic and electrostatic interactions, the pattern of these interactions is disparate (Figures 4B and 4C) casting doubt on their evolutionary relatedness. This, together with poor conservation of solvent-exposed residues, may indicate that the similarity between the CsfB and ClpX NTD dimers is limited to their structural folds, and does not extend to binding partners and/or function.

CsfB as an Anti-sigma Factor Structure

To our knowledge, CsfB is only the third anti-sigma factor for class III alternative sigma factors to be structurally analyzed, with the other two being the *Bacillus stereothermophilus* σ^F sporulation anti-sigma factor SpoIIAB (Campbell et al., 2002; Masuda et al., 2004) and the *Aquifex aeolicus* σ^{28} flagellum biosynthesis anti-sigma factor FlgM (Sorenson et al., 2004). CsfB is unrelated to both of these proteins, although SpoIIAB also homodimerizes (Campbell and Darst, 2000; Campbell et al., 2002). Aside from the ability to coordinate Zn^{2+}, CsfB also displays no structural similarity to the zinc anti-sigma (ZAS) family of anti-sigma factors that inhibit class IV ECF alternative sigma factors (Campbell et al., 2007; Sineva et al., 2017). Lastly, we had previously noted that CsfB resembles the *B. subtilis* σ^F inhibitor Fin at the primary amino acid sequence level (Camp et al., 2011). However, comparison of the CsfB structure with our recently reported Fin structure reveals that, although both proteins bind zinc, they fold into completely different motifs (Wang Erickson et al., 2017).

Sigma Factor Inhibition by CsfB

Our ultimate objective is a complete understanding of the mechanism by which CsfB inhibits σ^G and σ^E during

B. subtilis sporulation, and the regulation thereof. This study made significant headway toward this goal. We have shown that CsfB forms a tight homodimer that binds tightly to its two target sigma factors. In the case of the CsfB-σ^E interaction, our ITC analysis indicated a stoichiometry of 1:1, suggesting that a CsfB dimer simultaneously binds two σ^E molecules. We find the alternative scenario, in which the CsfB dimer dissociates upon binding σ^E, to be unlikely given that the CsfB HSQC spectrum does not drastically reconfigure upon addition of σ^E. Whether σ^G is bound in a similar manner remains an open question, given that we could neither calculate a stoichiometry from our ITC data nor observe the CsfB bound state by NMR.

Our data do not yet allow us to draw precise conclusions regarding the interface of CsfB that mediates contact with σ^E or σ^G. That said, it is evident that the C-terminal region of CsfB is required for interaction with both sigma factors *in vitro* and their inhibition *in vivo*. Our CSP analysis further indicates that this C-terminal region of CsfB becomes more structured upon interaction with σ^E. We therefore speculate that, at least for σ^E, two sigma factors are bound, one apiece, to the two C-terminal "tails" of a CsfB dimer. Interestingly, evidence in the literature suggests that the interaction of CsfB with σ^G is likely to be dissimilar, at least in its detail. CsfB binds to σ^G at region 2.1 while it binds to σ^E at regions 2.2–2.3. Moreover, specific amino acids that help CsfB discriminate between σ^F and σ^G play no role in discriminating between σ^E and σ^K, and vice versa (Serrano et al., 2011, 2015). As such, comparing and contrasting the structural basis for σ^G and σ^E inhibition by CsfB is an exciting challenge for future work.

Last but not least, it is tempting to speculate that degradation/cleavage of CsfB, which posed a significant challenge in this study, may be physiologically relevant in *B. subtilis*. For example, cleavage/degradation of CsfB may provide a mechanism by which σ^G ultimately escapes CsfB inhibition at late times in the forespore.

STAR★METHODS

Detailed methods are provided in the online version of this paper and include the following:

- KEY RESOURCES TABLE
- CONTACT FOR REAGENT AND RESOURCE SHARING
- EXPERIMENTAL MODEL AND SUBJECT DETAILS
- METHOD DETAILS
  - Plasmid Construction
  - *B. subtilis* Strain Construction
  - Recombinant Protein Production
  - NMR Spectroscopy
  - Structure Calculation
  - NMR Titrations
  - ITC
  - *In Vivo* σ^G and σ^E Inhibition Assay
- QUANTIFICATION AND STATISTICAL ANALYSIS
  - ITC Analysis
  - *In Vivo* σ^G and σ^E Inhibition Assay
- DATA AND SOFTWARE AVAILABILITY

SUPPLEMENTAL INFORMATION

Supplemental Information includes five figures and five tables and can be found with this article online at https://doi.org/10.1016/j.str.2018.02.007.

ACKNOWLEDGMENTS

R.L.I. was supported by MRC New Investigator Research Grant G0900936 and BBSRC grants BB/L006952/1 and BB/N006267/1. A.H.C. was supported by NIH grants DP2 GM105439 and R15 GM101559. NMR experiments were performed at the Centre for Biomolecular Spectroscopy, King's College London, established with a Capital Award from the Wellcome Trust. This work was supported by the Francis Crick Institute through provision of access to the MRC Biomedical NMR Centre, which receives its core funding from Cancer Research UK (FC001029), the UK MRC (FC001029), and the Wellcome Trust (FC001029). The 950 MHz NMR facility at the University of Oxford was funded by the Wellcome Trust Joint Infrastructure Fund and the E. P. Abraham Fund. We thank Dr J.M. Pérez-Cañadillas (Rocasolano Physical Chemistry Institute, Madrid, Spain) for providing a modified version of the pET28 vector and plasmid-encoding TEV protease. We also thank Dr Pete Simpson and Dr Geoff Kelly for NMR advice and Tanya Karagiannis for constructing the plasmid pTK2.

AUTHOR CONTRIBUTIONS

S.M.L., C.A., N.J.E., K.M.C., K.A.F., E.M.K., A.H.C., and R.L.I. conceived the ideas and designed experiments. S.M.L., C.A., N.J.E., K.M.C., K.A.F., R.A.A., E.M.K., A.V., J.J., and S.F.-O. performed experiments. S.M.L., C.A., N.J.E., K.M.C., R.A.A., E.M.K., A.H.C., and R.L.I. analyzed data. S.M.L., C.A., N.J.E., K.M.C., R.A.A., E.M.K., A.H.C., and R.L.I. contributed toward writing the manuscript.

DECLARATION OF INTERESTS

The authors declare no competing interests.

Received: September 26, 2017
Revised: November 17, 2017
Accepted: February 6, 2018
Published: March 8, 2018

SUPPORTING CITATIONS

The following references appear in the Supplemental Information: Fellinger et al. (2008); Markert et al. (2003); Pace and Scholtz (1998).

REFERENCES

Aslanidis, C., and de Jong, P.J. (1990). Ligation-independent cloning of PCR products (LIC-PCR). Nucleic Acids Res. *18*, 6069–6074.

Baker, T.A., and Sauer, R.T. (2012). ClpXP, an ATP-powered unfolding and protein-degradation machine. Biochim. Biophys. Acta *1823*, 15–28.

Camp, A.H., and Losick, R. (2008). A novel pathway of intercellular signalling in *Bacillus subtilis* involves a protein with similarity to a component of type III secretion channels. Mol. Microbiol. *69*, 402–417.

Camp, A.H., Wang, A.F., and Losick, R. (2011). A small protein required for the switch from σ^F to σ^G during sporulation in *Bacillus subtilis*. J. Bacteriol. *193*, 116–124.

Campagne, S., Allain, F.H., and Vorholt, J.A. (2015). Extra cytoplasmic function sigma factors, recent structural insights into promoter recognition and regulation. Curr. Opin. Struct. Biol. *30*, 71–78.

Campbell, E.A., and Darst, S.A. (2000). The anti-sigma factor SpoIIAB forms a 2:1 complex with σ^F, contacting multiple conserved regions of the σ factor. J. Mol. Biol. *300*, 17–28.

Campbell, E.A., Greenwell, R., Anthony, J.R., Wang, S., Lim, L., Das, K., Sofia, H.J., Donohue, T.J., and Darst, S.A. (2007). A conserved structural module

regulates transcriptional responses to diverse stress signals in bacteria. Mol. Cell *27*, 793–805.

Campbell, E.A., Masuda, S., Sun, J.L., Muzzin, O., Olson, C.A., Wang, S., and Darst, S.A. (2002). Crystal structure of the *Bacillus stearothermophilus* anti-σ factor SpoIIAB with the sporulation sigma factor σ^F. Cell *108*, 795–807.

Chary, V.K., Xenopoulos, P., and Piggot, P.J. (2007). Expression of the σ^F-directed *csfB* locus prevents premature appearance of σ^G activity during sporulation of *Bacillus subtilis*. J. Bacteriol. *189*, 8754–8757.

Decatur, A., and Losick, R. (1996). Identification of additional genes under the control of the transcription factor σ^F of *Bacillus subtilis*. J. Bacteriol. *178*, 5039–5041.

Delaglio, F., Grzesiek, S., Vuister, G.W., Zhu, G., Pfeifer, J., and Bax, A. (1995). NMRPipe: a multidimensional spectral processing system based on UNIX pipes. J. Biomol. NMR *6*, 277–293.

Donaldson, L.W., Wojtyra, U., and Houry, W.A. (2003). Solution structure of the dimeric zinc binding domain of the chaperone ClpX. J. Biol. Chem. *278*, 48991–48996.

Feklistov, A., Sharon, B.D., Darst, S.A., and Gross, C.A. (2014). Bacterial sigma factors: a historical, structural, and genomic perspective. Annu. Rev. Microbiol. *68*, 357–376.

Fellinger, K., Leonhardt, H., and Spada, F. (2008). A mutagenesis strategy combining systematic alanine scanning with larger mutations to study protein interactions. Anal. Biochem. *373*, 176–178.

Ferguson, C.C., Camp, A.H., and Losick, R. (2007). *gerT*, a newly discovered germination gene under the control of the sporulation transcription factor σ^K in *Bacillus subtilis*. J. Bacteriol. *189*, 7681–7689.

Gibson, D.G. (2009). Synthesis of DNA fragments in yeast by one-step assembly of overlapping oligonucleotides. Nucleic Acids Res. *37*, 6984–6990.

Grishin, N.V. (2001). Treble clef finger - a functionally diverse zinc-binding structural motif. Nucleic Acids Res. *29*, 1703–1714.

Grzesiek, S., and Bax, A. (1993). Amino acid type determination in the sequential assignment procedure of uniformly $^{13}C/^{15}N$-enriched proteins. J. Biomol. NMR *3*, 185–204.

Guérout-Fleury, A.M., Frandsen, N., and Stragier, P. (1996). Plasmids for ectopic integration in *Bacillus subtilis*. Gene *180*, 57–61.

Holm, L., and Laakso, L.M. (2016). Dali server update. Nucleic Acids Res. *44*, W351–W355.

Holm, L., and Rosenstrom, P. (2010). Dali server: conservation mapping in 3D. Nucleic Acids Res. *38*, W545–W549.

Karmazyn-Campelli, C., Rhayat, L., Carballido-Lopez, R., Duperrier, S., Frandsen, N., and Stragier, P. (2008). How the early sporulation sigma factor σ^F delays the switch to late development in *Bacillus subtilis*. Mol. Microbiol. *67*, 1169–1180.

Kaur, G., and Subramanian, S. (2016). Classification of the treble clef zinc finger: noteworthy lessons for structure and function evolution. Sci. Rep. *6*, 32070.

Kay, L.E., Xu, G.Y., Singer, A.U., Muhandiram, D.R., and Formankay, J.D. (1993). A gradient-enhanced HCCH TOCSY experiment for recording side-chain ^1H and ^{13}C correlations in H_2O samples of proteins. J. Magn. Reson. B *101*, 333–337.

Koradi, R., Billeter, M., and Wuthrich, K. (1996). MOLMOL: a program for display and analysis of macromolecular structures. J. Mol. Graph. *14*, 51–55, 29–32.

Kornhaber, G.J., Snyder, D., Moseley, H.N., and Montelione, G.T. (2006). Identification of zinc-ligated cysteine residues based on $^{13}C\alpha$ and $^{13}C\beta$ chemical shift data. J. Biomol. NMR *34*, 259–269.

Krishna, S.S., Majumdar, I., and Grishin, N.V. (2003). Structural classification of zinc fingers. Nucleic Acids Res. *31*, 532–550.

Krissinel, E., and Henrick, K. (2007). Inference of macromolecular assemblies from crystalline state. J. Mol. Biol. *372*, 774–797.

Markert, Y., Koditz, J., Ulbrich-Hofmann, R., and Arnold, U. (2003). Proline versus charge concept for protein stabilization against proteolytic attack. Protein Eng. *16*, 1041–1046.

Masuda, S., Murakami, K.S., Olson, S.W.C.A., Donigian, J., Leon, F., Darst, S.A., and Campbell, E.A. (2004). Crystal structures of the ADP and ATP bound forms of the *Bacillus* anti-sigma factor SpoIIAB in complex with the anti-anti-sigma SpoIIAA. J. Mol. Biol. *340*, 941–956.

Osterberg, S., del Peso-Santos, T., and Shingler, V. (2011). Regulation of alternative sigma factor use. Annu. Rev. Microbiol. *65*, 37–55.

Pace, C.N., and Scholtz, J.M. (1998). A helix propensity scale based on experimental studies of peptides and proteins. Biophys. J. *75*, 422–427.

Paget, M.S. (2015). Bacterial sigma factors and anti-sigma factors: structure, function and distribution. Biomolecules *5*, 1245–1265.

Park, E.Y., Lee, B.G., Hong, S.B., Kim, H.W., Jeon, H., and Song, H.K. (2007). Structural basis of SspB-tail recognition by the zinc binding domain of ClpX. J. Mol. Biol. *367*, 514–526.

Peters, H.K., 3rd, Carlson, H.C., and Haldenwang, W.G. (1992). Mutational analysis of the precursor-specific region of *Bacillus subtilis* σ^E. J. Bacteriol. *174*, 4629–4637.

Rhayat, L., Duperrier, S., Carballido-Lopez, R., Pellegrini, O., and Stragier, P. (2009). Genetic dissection of an inhibitor of the sporulation sigma factor σ^G. J. Mol. Biol. *390*, 835–844.

Rieping, W., Habeck, M., Bardiaux, B., Bernard, A., Malliavin, T.E., and Nilges, M. (2007). ARIA2: automated NOE assignment and data integration in NMR structure calculation. Bioinformatics *23*, 381–382.

Schmalisch, M., Maiques, E., Nikolov, L., Camp, A.H., Chevreux, B., Muffler, A., Rodriguez, S., Perkins, J., and Losick, R. (2010). Small genes under sporulation control in the *Bacillus subtilis* genome. J. Bacteriol. *192*, 5402–5412.

Serrano, M., Gao, J., Bota, J., Bate, A.R., Meisner, J., Eichenberger, P., Moran, C.P., Jr., and Henriques, A.O. (2015). Dual-specificity anti-sigma factor reinforces control of cell-type specific gene expression in *Bacillus subtilis*. PLoS Genet. *11*, e1005104.

Serrano, M., Real, G., Santos, J., Carneiro, J., Moran, C.P., Jr., and Henriques, A.O. (2011). A negative feedback loop that limits the ectopic activation of a cell type-specific sporulation sigma factor of *Bacillus subtilis*. PLoS Genet. *7*, e1002220.

Shen, Y., Delaglio, F., Cornilescu, G., and Bax, A. (2009). TALOS+: a hybrid method for predicting protein backbone torsion angles from NMR chemical shifts. J. Biomol. NMR *44*, 213–223.

Sineva, E., Savkina, M., and Ades, S.E. (2017). Themes and variations in gene regulation by extracytoplasmic function (ECF) sigma factors. Curr. Opin. Microbiol. *36*, 128–137.

Sorenson, M.K., Ray, S.S., and Darst, S.A. (2004). Crystal structure of the flagellar σ/anti-σ complex σ^{28}/FlgM reveals an intact σ factor in an inactive conformation. Mol. Cell *14*, 127–138.

Steinmetz, M., and Richter, R. (1994). Plasmids designed to alter the antibiotic resistance expressed by insertion mutations in *Bacillus subtilis*, through in vivo recombination. Gene *142*, 79–83.

Tan, I.S., and Ramamurthi, K.S. (2014). Spore formation in *Bacillus subtilis*. Environ. Microbiol. Rep. *6*, 212–225.

Thibault, G., Yudin, J., Wong, P., Tsitrin, V., Sprangers, R., Zhao, R.M., and Houry, W.A. (2006). Specificity in substrate and cofactor recognition by the N-terminal domain of the chaperone ClpX. Proc. Natl. Acad. Sci. USA *103*, 17724–17729.

Vranken, W.F., Boucher, W., Stevens, T.J., Fogh, R.H., Pajon, A., Llinas, M., Ulrich, E.L., Markley, J.L., Ionides, J., and Laue, E.D. (2005). The CCPN data model for NMR spectroscopy: development of a software pipeline. Proteins *59*, 687–696.

Wach, A. (1996). PCR-synthesis of marker cassettes with long flanking homology regions for gene disruptions in *S. cerevisiae*. Yeast *12*, 259–265.

Wang Erickson, A.F., Deighan, P., Chen, S.S., Barrasso, K., Garcia, C.P., Martinez-Lumbreras, S., Alfano, C., Krysztofinska, E.M., Thapaliya, A.,

Camp, A.H., et al. (2017). A novel RNA polymerase-binding protein that interacts with a sigma-factor docking site. Mol. Microbiol. *105*, 652–662.

Wilson, G.A., and Bott, K.F. (1968). Nutritional factors influencing development of competence in *Bacillus subtilis* transformation system. J. Bacteriol. *95*, 1439–1449.

Youngman, P., Perkins, J.B., and Losick, R. (1984). Construction of a cloning site near one end of Tn917 into which foreign DNA may be inserted without affecting transposition in *Bacillus subtilis* or expression of the transposon-borne *erm* gene. Plasmid *12*, 1–9.

Zwahlen, C., Legault, P., Vincent, S.J.F., Greenblatt, J., Konrat, R., and Kay, L.E. (1997). Methods for measurement of intermolecular NOEs by multinuclear NMR spectroscopy: application to a bacteriophage λ N-peptide/*boxB* RNA complex. J. Am. Chem. Soc. *119*, 6711–6721.

STAR★METHODS

KEY RESOURCES TABLE

REAGENT or RESOURCE	SOURCE	IDENTIFIER
Bacterial and Virus Strains		
E. coli BL21(DE3)pLysS	New England Biolabs	Cat# C2527I
E. coli NEB5-alpha	New England Biolabs	Cat# C2987I
Chemicals, Peptides, and Recombinant Proteins		
Ampicillin	Melford Laboratories, Sigma-Aldrich	Cat# A0104, Cat# A9518
Kanamycin	Melford Laboratories, Sigma-Aldrich	Cat# K0126, Cat# K4000
Chloramphenicol	Sigma Aldrich	Cat# C0378
Erythromycin	Sigma-Aldrich	Cat# E6376
Lincomycin	VWR (Alfa Aesar)	Cat #AAJ61251
Spectinomycin	Sigma-Aldrich	Cat# S9007
Phleomycin	Research Products International	Cat# P20200
Tetracycline	VWR (Alfa Aesar)	Cat# AAB21408
LB Broth	Research Products International	Cat# L24061, Cat# L24065
LB Broth High Salt	Melford Laboratories	Cat# L1704
Agar, Bacteriological Grade	VWR (Hardy Diagnostics)	Cat# 89405-068
IPTG	Sigma-Aldrich, Research Products International	Cat# I6758, Cat# AAJ61251
$ZnCl_2$	Sigma-Aldrich	Cat# 229997
^{15}N-NH_4Cl	Sigma-Aldrich	Cat# 299251
^{13}C-glucose	Sigma-Aldrich	Cat# 389374
^{13}C-^{15}N-Isogro	Sigma-Aldrich	Cat# 606839
Deuterium Oxide	Sigma-Aldrich	Cat# 151882
HEPES	Melford Laboratories	Cat# B2001
NaCl	Melford Laboratories	Cat# S0520
$MgCl_2$	Melford Laboratories	Cat# M0535
KCl	Melford Laboratories	Cat# P0515
Imidazole	Melford Laboratories	Cat# B4005
cOmplete mini EDTA-free protease inhibitor tablets	Roche	Cat# 11836170001
TCEP	Alfa Aesar	Cat# J60316.09
DTT	Sigma-Aldrich	Cat# D0632
PMSF	Sigma-Aldrich	Cat# P7626
Glycerol	VWR	Cat# 24388.295
DNAse I grade II	Roche	Cat# 10104159001
Lysozyme	Sigma Aldrich	Cat# L6876
Critical Commercial Assays		
Q5 Site-Directed Mutagenesis Kit	New England Biolabs	Cat# E0554S
QuikChange Mutagenesis Kit	Agilent Technologies	Cat# 200517
Gibson Assembly Master Mix	New England Biolabs	Cat# E2611S
NEBuilder HiFi DNA Assembly Master Mix	New England Biolabs	Cat# E2621S
Deposited Data		
Solution structure of CsfB 1-48	This study	PDB: 5N7Y
Chemical shift assignment of CsfB 1-48	This study	BMRB: 34102
Experimental Models: Organisms/Strains		
B. subtilis: Parent strain PY79	Youngman et al., 1984	N/A
B. subtilis: Strain AHB98 (*ΔsigG::kan*)	Camp and Losick, 2008	N/A

(*Continued on next page*)

Continued

REAGENT or RESOURCE	SOURCE	IDENTIFIER
B. subtilis: Strain AHB199 (*ΔcsfB*::*tet*)	Camp and Losick, 2008	N/A
B. subtilis: Strain AHB201 (*ΔsigE*::*erm*)	This study	N/A
B. subtilis: Strain SFB31 (*ΔsigE*::[*erm*]::*phleo*)	This study	N/A
B. subtilis: Strains used for *in vivo* σ^G and σ^E inhibition assays, see Table S5	This study	N/A
Oligonucleotides		
Primers used for plasmid construction, see Table S4	Integrated DNA Technologies	N/A
Synthetic gene fragments used for plasmid construction	Integrated DNA Technologies	N/A
Recombinant DNA		
Plasmid: pET-46	Novagen	Cat# 71335-3
Plasmid: pNIC28	Structural Genomics Consortium	Cat# 26103
Plasmid: pLATE31	Thermo Scientific	Cat# K1261
Plasmid: pET28_TxrA	José Manuel Pérez Cañadillas	N/A
Plasmid: pDR110	David Rudner	N/A
Plasmid: pDR111	David Rudner	N/A
Plasmid: pDG1664	Guérout-Fleury et al., 1996	N/A
Plasmid: pAH321	Schmalisch et al., 2010	N/A
Plasmid: pAH328	This study	N/A
Plasmid: pEr::Pm	Steinmetz and Richter, 1994	N/A
Plasmids constructed for expression of CsfB, σ^G, or σ^E in *E. coli* or *B. subtilis*, see Table S3	This study	N/A
Plasmids harboring σ^G- or σ^E-dependent luciferase reporters, see Table S3	This study	N/A
Software and Algorithms		
Topspin 3	Bruker Biospin	https://www.bruker.com/service/support-upgrades/software-downloads/nmr.html
NMRPipe/NMRDraw	Delaglio et al., 1995	http://www.nmrpipe.com/
CcpNMR Analysis 2.2	Vranken et al., 2005	http://www.ccpn.ac.uk/v2-software/software/analysis
ARIA2.3	Rieping et al., 2007	http://aria.pasteur.fr/downloads
TALOS+	Shen et al., 2009	https://spin.niddk.nih.gov/bax/software/TALOS/
MOLMOL	Koradi et al., 1996	http://www.msg.ucsf.edu/local/programs/molmol/manual.html
PyMOL	DeLano Scientific LLC	http://www.pymol.org
MicroCal Origin 7	OriginLab	https://www.originlab.com/
Gen5 Microplate Reader and Imager Software	BioTek Instruments	https://www.biotek.com/
Excel	Microsoft Corporation	https://office.microsoft.com/excel/
Prism	GraphPad Software	https://www.graphpad.com/
Other		
HisTrap FF crude column pre-packed with Ni Sepharose resin	GE Healthcare Life Sciences	Cat# 17-5286-01
Superdex75 16/60 PG column	GE Healthcare Life Sciences	Cat# 17-1068-01

CONTACT FOR REAGENT AND RESOURCE SHARING

Further information and requests for resources and reagents should be directed to and will be fulfilled by the Lead Contact, Rivka Isaacson (rivka.isaacson@kcl.ac.uk).

EXPERIMENTAL MODEL AND SUBJECT DETAILS

In vivo functionality tests of CsfB variants were performed in *B. subtilis* strains isogenic with the laboratory strain PY79 (Youngman et al., 1984). For general propagation, *B. subtilis* strains were grown at 37°C in liquid LB media or on LB agar plates.

METHOD DETAILS

Plasmid Construction

Plasmids used in this study are listed in the Key Resources Table and Table S3. The sequences of oligonucleotides used in plasmid construction are given in Table S4. Chromosomal DNA from *B. subtilis* strain PY79 served as a template for polymerase chain reaction (PCR), unless otherwise noted. Sequences of synthetic gene fragments (gBlocks, Integrated DNA Technologies) used in plasmid construction are available upon request. Plasmids were constructed using traditional cloning techniques, site-directed mutagenesis, ligation-independent cloning (Aslanidis and de Jong, 1990), or isothermal assembly (Gibson, 2009), as indicated. Plasmids were propagated in the *E. coli* strain NEB 5-alpha grown in the presence of the antibiotics ampicillin (100 μg/mL) or kanamycin (50 μg/mL), when appropriate. Site-directed mutagenesis was performed either with the Q5 Site-Directed Mutagenesis Kit or QuikChange Mutagenesis Kit. Isothermal assembly was performed with either the Gibson Assembly Master Mix or the NEBuilder HiFi DNA Assembly Master Mix. All plasmids were verified by DNA sequencing. Construction details for plasmids not previously published are given below.

To generate plasmids for expression and purification of recombinant CsfB from *E. coli*, the *csfB* coding sequence was amplified by PCR using primers listed in Table S4 and inserted by ligation independent cloning into pET-46 (N-terminal hexahistidine tag), pNIC28 (TEV protease-cleavable N-terminal hexahistidine tag), and pLATE31 (C-terminal hexahistidine tag), respectively. Plasmids encoding $CsfB^{A48E}$ and other CsfB variants were obtained by site-directed mutagenesis of the pNIC28-CsfB plasmid using the Q5 Site-Directed Mutagenesis Kit.

The plasmid for expression and purification of recombinant σ^G from *E. coli* was generated by ligation independent cloning of a PCR product harboring the full-length *sigG* coding sequence into pET-46 (N-terminal hexahistidine tag). The plasmid for expression and purification of σ^E (lacking its N-terminal membrane-anchored pro-sequence) from *E. coli* was constructed by ligating a BamHI/XhoI-digested PCR product harboring *sigE* codons 17-239 into BamHI/XhoI-digested pET28-TxrA (gift of Dr J.M. Pérez-Cañadillas, Rocasolano Physical Chemistry Institute, Spain), an *E. coli* expression plasmid containing a TEV protease cleavable N-terminal hexahistidine and thioredoxin tags.

To construct the plasmid (pJJ46) for induction of σ^G expression in *B. subtilis*, the *sigG* 5' leader (including the native *sigG* ribosome binding site [RBS]) and coding sequence was amplified by PCR with primers JJ30 and JJ31 and assembled into SalI/NheI-digested pDR110 (gift of David Rudner, Harvard Medical School). The plasmid (pSFO1) for induction of σ^E (lacking its N-terminal membrane-anchored pro-sequence) in *B. subtilis* was constructed by assembling a synthetic gene fragment harboring *sigE* codons 17-239 preceded by an optimized RBS into SalI/SphI-digested pDR111 (gift of David Rudner, Harvard Medical School). The plasmid (pAH88) for IPTG-induction of CsfB in *B. subtilis* was generated in two steps. First, the *csfB* 5' leader (including the native RBS) and coding sequence were amplified using primers AH41 and AH42, digested with HindIII and NheI, and cloned into HindIII/NheI-digested pDR111 (gift of David Rudner, Harvard Medical School), yielding the intermediate plasmid pAH84. The EcoRI/BamHI fragment containing P_{spank}-*csfB* and *lacI* was then subcloned into the respective sites of pDG1664 (Guérout-Fleury et al., 1996) to generate pAH88. The derivatives of pAH88 encoding CsfB variants A48E, V37A, V37E, I38A, I38E, V37A/I38A, or the $CsfB^{1-48}$ truncation (pKF70, pKF87-pKF91, and pTK2, respectively) were constructed by individually assembling synthetic gene fragments into the HindIII/SphI-digested pAH88 backbone.

Plasmids encoding luciferase reporter genes were constructed using the plasmid pAH328, which harbors the *Photorhabdus luminescens* bacterial luciferase operon *luxABCDE* optimized for expression in *B. subtilis* and preceded by a multiple cloning site (MCS). This plasmid was constructed from pAH321 (Schmalisch et al., 2010) in two steps. First, the BamHI site upstream of the *luxE* coding sequence in pAH321 was mutated by site-directed mutagenesis using the QuikChange Mutagenesis Kit and primers AH312 and AH313, yielding pAH325. Second, a DNA fragment harboring an EcoRI-SacI-NotI-SpeI-SalI MCS, generated by annealing oligonucleotides AH310 and AH311, was ligated into the EcoRI/SalI-digested backbone of pAH325, yielding pAH328. The σ^E- and σ^G-dependent luciferase reporter plasmids (pAH334 and pAH336, respectively) were constructed by ligating EcoRI/SalI-digested PCR products containing either the σ^E-dependent *spoIID* promoter (amplified with AH58 and AH59) or the σ^G-dependent *sspB* promoter (amplified with AH60 and AH61) into EcoRI/SalI-digested pAH328.

B. subtilis Strain Construction

The full genotypes of *B. subtilis* strains used in this study, all of which were derived from the wild type laboratory strain PY79 (Youngman et al., 1984), are listed in the Key Resources Table and Table S5. Strains were constructed by transformation of competent cells, prepared as previously described (Wilson and Bott, 1968), with *B. subtilis* chromosomal DNA, plasmid DNA, or PCR-amplified DNA. Transformants were selected on media with antibiotics, when appropriate, as follows: chloramphenicol (5 μg/mL), erythromycin plus lincomycin (1 μg/mL and 25 μg/mL, respectively), spectinomycin (100 μg/mL), kanamycin (5 μg/mL), phleomycin (0.4 μg/mL), and tetracycline (10 μg/mL). Insertions into *amyE* or *thrC* were confirmed by loss of α-amylase activity on LB agar plates with starch or the failure to grow on minimal media, respectively.

The Δ*csfB*::*tet* (AHB199) and Δ*sigG*::*kan* (AHB98) deletions have been described (Camp and Losick, 2008). The Δ*sigE*::[*erm*]::*phleo* deletion was built for this study in two steps. First, a Δ*sigE*::*erm* deletion strain (AHB201) was constructed by the long-flanking homology PCR (LFH-PCR) method (Wach, 1996). Primers sets AH43/AH44 and AH45/AH46 were used to amplify sequences flanking *sigE*, which were then used to amplify the erythromycin resistance cassette (*erm*) from plasmid pAH52 (Ferguson et al., 2007). Proper integration of the resulting Δ*sigE*::*erm* LFH-PCR product was confirmed by PCR. To switch the antibiotic resistance of the Δ*sigE* deletion, AHB201 was transformed with pEr::Pm (Steinmetz and Richter, 1994), resulting in the erythromycin-sensitive, phleomycin-resistant strain SFB31 (Δ*sigE*::[*erm*]::*phleo*).

All other constructs (IPTG-inducible *sigG*, *sigE*, and *csfB*, as well as the σ^E- and σ^G-dependent luciferase reporters) were introduced into *B. subtilis* strains using plasmids constructed for this study.

Recombinant Protein Production

The plasmids encoding CsfB, σ^G or σ^E (see the Key Resources Table and Table S3) were transformed into the BL21(DE3)pLysS *E. coli* strain. Cells were grown either in LB or minimal media supplemented with 0.7 g/l ^{15}N-NH_4Cl, 2 g/l ^{13}C-glucose and 1 g/l ^{13}C-^{15}N-Isogro. Protein expression was induced with 0.5 mM IPTG at OD_{600} = 0.8 and conducted either at 37°C for 4 hours or at 18°C overnight. $ZnCl_2$ at a final concentration of 10 μM was added to minimal media cell culture before IPTG induction for production of CsfB.

The cell pellet was resuspended in 50 mM HEPES pH 7.5, 300 mM NaCl, 5 mM Imidazole, 5% Glycerol, 1 mM DTT, 1 mg/ml lysozyme, 10 μg/ml Dnase I, 5 mM $MgCl_2$, 3x EDTA-free Complete Protease Inhibitor and 2 mM phenylmethylsulfonyl fluoride (PMSF), then lysed by sonication. Recombinant protein was purified from the soluble fraction of the cell lysate by affinity chromatography using a ready-to-use HisTrap FF crude column pre-packed with Ni Sepharose resin. When required, the N-terminal His-tag was removed by overnight incubation at 4°C with TEV protease at a molar ratio protein:TEV of 40:1. The digested protein was then separated from undigested protein and TEV protease using HisTrap FF crude column pre-packed with Ni Sepharose resin. Purified fractions were subjected to a final step of size exclusion chromatography using a Superdex75 16/600 PG column equilibrated with 50 mM HEPES pH 7.5, 150 mM KCl and 0.5 mM TCEP buffer.

The purity and stability of the proteins were checked by SDS-PAGE and mass spectrometry and the presence and stoichiometry of zinc in CsfB was determined by Inductively Coupled Plasma Mass Spectrometry (ICP-MS) using a PerkinElmer NexION 350D spectrometer.

NMR Spectroscopy

Uniformly ^{15}N, ^{13}C-labelled NMR sample was buffer-exchanged into 50 mM HEPES pH 7.5, 150 mM KCl, 0.5 mM TCEP using a HiLoad 16/600 Superdex 75 pg gel filtration column. NMR experiments were carried out on samples >500 μM at 303 K and recorded on Bruker AVANCE spectrometers operating at 500 MHz, 700 MHz and 950 MHz with TXI cryoprobes controlled by Topspin 3 (Bruker Biospin Ltd). Spectra were processed using NMRPipe/NMRDraw (Delaglio et al., 1995) and analyzed using CcpNMR Analysis 2.2 (Vranken et al., 2005). Backbone resonances were assigned in a standard manner using [^{1}H,^{15}N]-HSQC, HNCA, HNCACB, CBCA(CO)NH, and HNCO experiments (Grzesiek and Bax, 1993). Side-chains resonances assignment was performed using a combination of HCCH-TOCSY (Kay et al., 1993) and HBHA(CO)NH (Grzesiek and Bax, 1993). NOE distance restraints and assignments of aromatics rings were obtained from ^{15}N-edited NOESY-HSQC and ^{13}C-edited NOESY-HSQC spectra with a 120 ms mixing time. An additional set of intermolecular distance restraints was obtained from a ^{12}C-filtered, ^{13}C-edited NOESY-HSQC spectrum (Zwahlen et al., 1997) using a mixed CsfB dimer prepared by mixing ^{15}N, ^{13}C-labelled CsfB and unlabelled CsfB in an equimolar ratio. To allow the exchange of the monomeric subunits, the mixture was heated at 50°C for 10 minutes and then cooled slowly.

Structure Calculation

The solution structure of the CsfB$^{1\text{-}48}$ dimer was solved using ARIA2.3 (Rieping et al., 2007), utilizing distance restraints derived from the four NOESY spectra (NOEs from the filtered NOESY experiment were defined as intermolecular while the NOEs in the other NOESY experiments were treated as ambiguous) and dihedral angle restraints estimated by TALOS+ (Shen et al., 2009). Typical annealing parameters were used for distance (10, 15, 50 and 100 Kcal/mol for high temperature, initial cool1, final cool1 and cool2 force constants) and dihedral restraints (50, 150 and 200 Kcal/mol for high temperature, cool1 and cool2 force constants) and a C2 symmetry was imposed with a non-crystallographic symmetry restraints force constant value of 100 Kcal/mol and packing force constants of 15, 10 and 5 Kcal/mol during high temperature, cool1 and cool2 steps.

In the first rounds of calculation, zinc coordination information was not included and, only after checking that the putative involved cysteine residues appeared at the correct disposition for tetrahedral coordination, were the appropriate restrictions for the zinc fingers added (using ARIA2.3 tools). Twenty structures with the lowest energy values were selected out of 200 and subjected to a water refinement process. The final ensemble of the structure (PDB: 5N7Y) was analyzed and represented using MOLMOL (Koradi et al., 1996) and PyMOL.

NMR Titrations

Chemical shift perturbation assays were carried out at 298K using a Bruker AVANCE spectrometer operating at 950 MHz with a TXI cryoprobe controlled by Topspin 3 (Bruker Biospin Ltd). Spectra were processed using NMRPipe/NMRDraw (Delaglio et al., 1995)

and analyzed using CcpNMR Analysis 2.2 (Vranken et al., 2005). 100 μM ^{15}N-labelled CsfB^{A48E} in 50 mM HEPES pH 7.5, 150 mM KCl, 0.5 mM TCEP was titrated with unlabelled σ^G or σ^E up to a ratio of 1:2 molar equivalents. ^{1}H–^{15}N SOFAST-HMQC spectra were recorded at each titration point.

ITC

Binding of CsfB^{A48E} to σ^G or σ^E was measured by ITC using an ITC200 instrument (Microcal Inc. Malvern). Samples were dialyzed into 50 mM HEPES pH 7.5, 150 mM KCl, 0.5 mM TCEP. Titrations were carried out at 25°C using 19 injections of 2 μl with a delay of 180s between injections. For the CsfB^{A48E} interaction with σ^G the sample cell contained 110 μM σ^G and the syringe 1.6 mM CsfB^{A48E}. For the CsfB^{A48E} interaction with σ^E the sample cell contained 60 μM CsfB^{A48E} and the syringe 395 μM σ^E.

In Vivo σ^G and σ^E Inhibition Assay

To measure CsfB-mediated σ^G and σ^E inhibition *in vivo*, *B. subtilis* strains harboring σ^G- or σ^E-dependent luciferase reporter genes were engineered to induce expression of the corresponding sigma factors either alone or in combination with wild type or mutant CsfB. Equal amounts of vegetatively growing cells (1 OD_{600}•mL) were collected and concentrated 5-fold. 30 μl of these cells were applied onto 200 μl LB agar pads containing 100 μM IPTG (for σ^G activity assays) or 10 μM IPTG (for σ^E activity assays) in white 96-well plates. Bioluminescence from each well was measured at 37°C every 15 min for 6 hours using a Synergy H1M plate reader (BioTek Instruments). Data is reported as the average of at least two (typically three or more) different experiments, with 2-4 technical replicates performed per experiment. CsfB inhibition was calculated as the percentage reduction in σ^G or σ^E activity (with background reporter activity subtracted) after 4 or 3 hours of induction, respectively, relative to the total σ^G or σ^E activity (also with background reporter activity subtracted) in a strain lacking inducible *csfB*.

QUANTIFICATION AND STATISTICAL ANALYSIS

ITC Analysis

The obtained data from ITC titrations were analyzed using MicroCal Origin 7 software. Areas under the peaks were integrated and fitted by least-square procedures assuming a 1:1 stoichiometry.

In Vivo σ^G and σ^E Inhibition Assay

Data obtained from the *in vivo* σ^G and σ^E inhibition assays was collected using the Gen5 Microplate Reader and Imager Software (BioTek Instruments) and subsequently analyzed using Excel (Microsoft Corporation) and Prism (Graphpad Software). Variation in the data was determined by calculating the standard deviation across separate experiments.

DATA AND SOFTWARE AVAILABILITY

The coordinates of the final ensemble of CsfB structure are deposited at the Protein Data Bank Europe (https://www.ebi.ac.uk/pdbe/) under the accession code 5N7Y. The assigned chemical shifts of the protein are also deposited at the Biological Magnetic Resonance Bank (http://www.bmrb.wisc.edu/) under the accession number 34102.

Structure, Volume *26*

Supplemental Information

Structural and Functional Insights into *Bacillus subtilis* Sigma Factor Inhibitor, CsfB

Santiago Martínez-Lumbreras, Caterina Alfano, Nicola J. Evans, Katherine M. Collins, Kelly A. Flanagan, R. Andrew Atkinson, Ewelina M. Krysztofinska, Anupama Vydyanath, Jacquelin Jackter, Sarah Fixon-Owoo, Amy H. Camp, and Rivka L. Isaacson

SUPPLEMENTAL FIGURES S1-S4

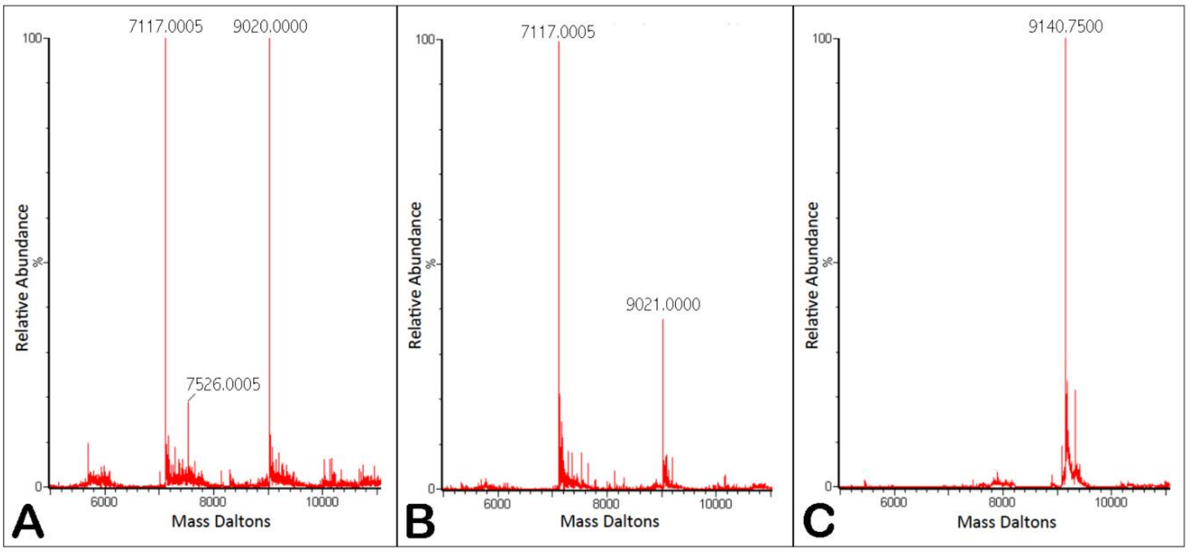

Figure S1 (related to Figure 1): Electrospray ionization mass spectrometry of CsfB. (A) N-terminally His-tagged CsfB immediately following size-exclusion chromatography purification displayed two distinct species with mass correlating to full length (9020.16 Da) and a 1-48 cleaved species (7116.86 Da) at a 50:50 ratio. **(B)** After storage at 4°C for four days the cleaved species represented 75% of the sample. **(C)** The CsfB^{A48E} variant was protected from degradation, remaining uncleaved even after storage at 4°C for four days.

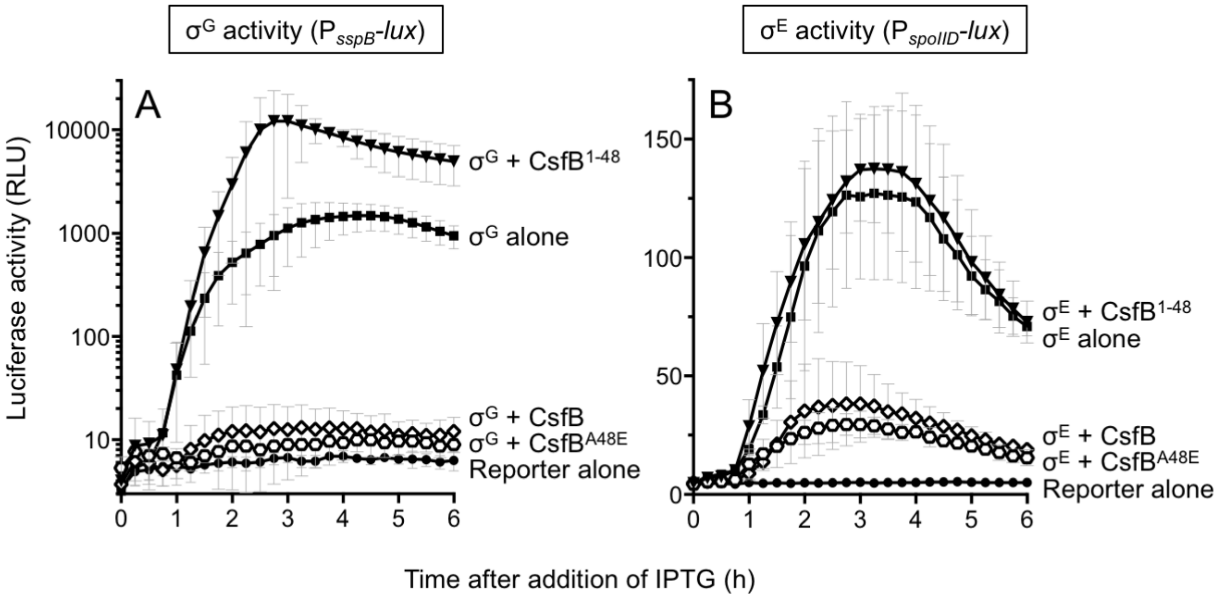

Figure S2 (related to Figure 1): Functionality of the CsfB$^{1\text{-}48}$ and CsfB^{A48E} variants *in vivo*. To assess the ability of CsfB$^{1\text{-}48}$ and CsfB^{A48E} to inhibit **(A)** σ^G or **(B)** σ^E *in vivo*, expression of each sigma factor was induced during vegetative growth either alone or in combination with wild type or variant CsfB proteins. Sigma factor activity following the addition of inducer (IPTG) was monitored every 15 minutes for 6 hours by light production (measured in relative light units [RLU]) from σ^G- or σ^E-dependent luciferase reporter genes (P_{sspB}-*lux* or P_{spoIID}-*lux*, respectively). Light production by strains expressing σ^G or σ^E alone or in combination with wild type CsfB, as well as a control strains without any inducible constructs ("Reporter alone") are shown for comparison in each graph. Data are reported as the average of at least two experiments, with error bars indicating standard deviation. Strains used in this assay are listed in Table S4.

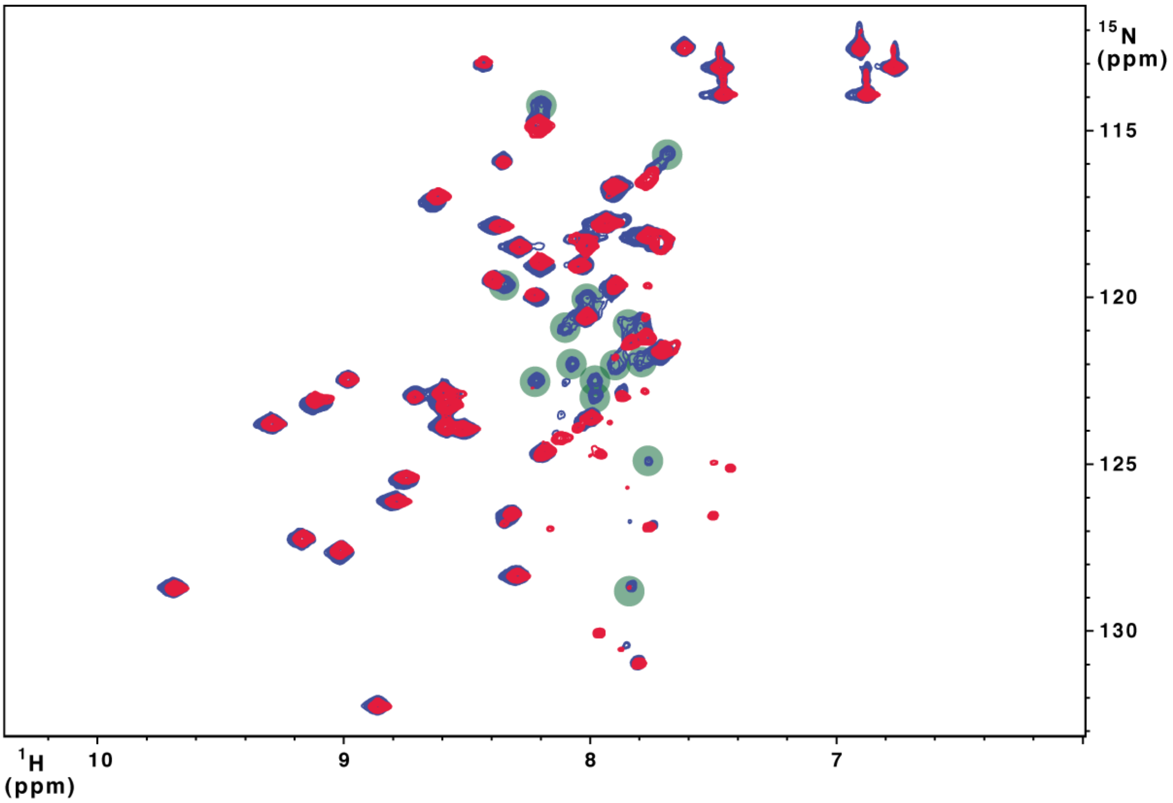

Figure S3 (Related to Figure 1): C-terminally His-tagged CsfB degradation can be observed by NMR spectroscopy. ^{1}H-^{15}N HSQC spectra of fresh protein immediately following purification (blue) and after two days (red). Shadowed peaks correspond to the unstable C-terminal residues of the protein.

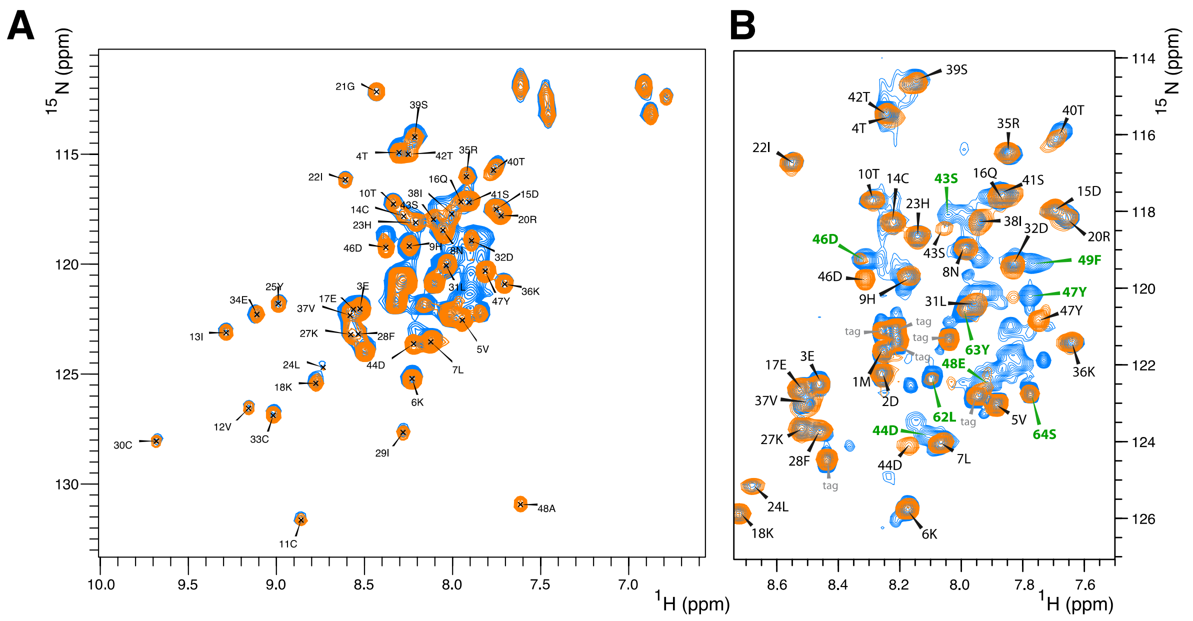

Figure S4 (Related to Figure 1): Wild type CsfB and CsfB^{A48E} observed by NMR spectroscopy. (A) ^{1}H-^{15}N SOFAST HMQC of CsfB (orange) overlaid onto CsfB^{A48E} (blue). Note the chemical shift conservation of the N-terminal folded domain and the appearance of new peaks at around 8ppm in proton dimension corresponding to the C-terminal region in CsfB^{A48E} spectrum (blue). **(B)** ^{1}H-^{15}N TROSY HSQC of CsfB^{WT} (orange) overlaid onto CsfB^{A48E} (blue) zoomed in on the central region of the spectra. Assigned peaks of CsfB are labelled in black. Extra or shifted peaks in the CsfB^{A48E} spectrum are labelled in green and peaks arising from the N-terminal His tag are labelled in grey.

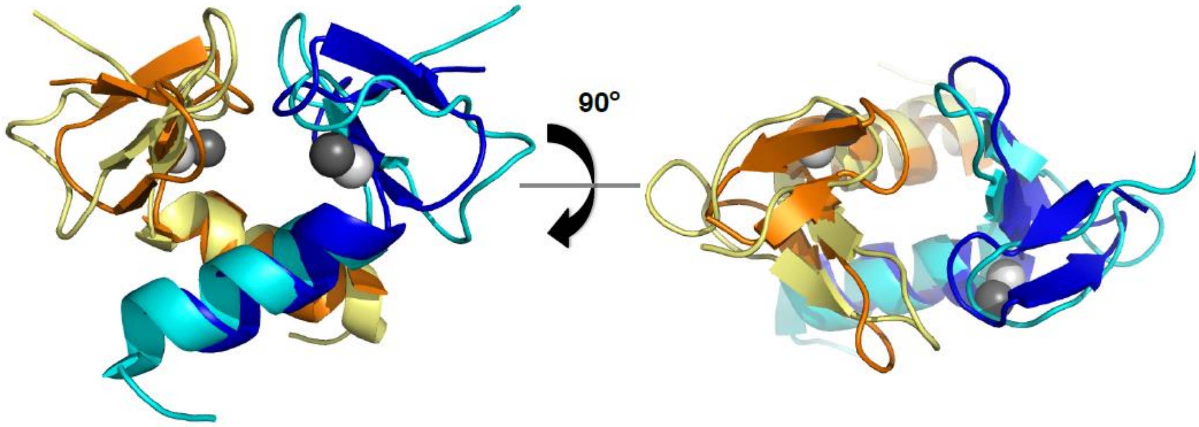

Figure S5 (Related to Figure 4): Structural alignment of CsfB and the ClpX N-terminal domain (NTD). The CsfB structure from this work (PDB: 5N7Y) is shown in orange and blue, and the ClpX NTD (PDB: 2DS6; (Park et al., 2007)) is shown in light yellow and cyan.

SUPPLEMENTAL TABLES S1-S5

Table S1 (Related to Figure 1). Protein engineering strategy to isolate a stabilised CsfB variant.

Mutation	Mutation Type	Expressed?	EI MS/NMR HSQC Results
A48P	Proline concept	Y	Stable full length protein
A48L	Conserved aliphatic	N	-
A48E	Charge principle	Y	Stable full length protein
F49A	Alanine substitution	Y	-
F49L	Conserved aliphatic	N	-
Y50F	Conserved bulk	Y	-
Y50E	Conserved charge	Y	-
K52A	Alanine substitution	Y	15% full length protein, 85% cleavage to residue 52
K53A	Alanine substitution	Y	-
K55A	Alanine substitution	Y	-

Two patches of conserved aromatic and basic residues near the C-terminus of CsfB, typical targets for trypsin-like and chymotrypsin-like proteases, were selected for mutation. Mutations were based on the proteolytic-halting charge principle and proline concept, as well as conservative mutations and classic alanine substitutions (Fellinger et al., 2008; Markert et al., 2003; Pace and Scholtz, 1998). Eight constructs were successfully expressed, but SDS and native PAGE gels from purification were inconclusive. Three constructs were taken forward to be assessed by electrospray ionization mass spectrometry (Figure S1) and NMR HSQC (Figure S4) for protein length and stability, two of which were found to be full-length and stable over time (A48P and A48E). CsfB^{A48E}, which was found to have comparable activity to wild-type CsfB *in vivo* (Figure S2), was used for future studies.

Table S2 (Related to Figure 3). Geometry of the zinc coordination centre of CsfB.

Averaged distance and angular values (for both monomers) for first and second coordination shell across the NMR ensemble.

	**Cys 11**	**Cys 14**	**Cys 30**	**Cys 33**
First coordination shell				
Sγ-Zn distance (Å)	2.30 ± 0.02	2.31 ± 0.01	2.29 ± 0.01	2.30 ± 0.01
Cβ-Sγ-Zn angle (º)	110.4 ± 4.5	111.6 ± 1.2	109.1 ± 1.1	110.7 ± 2.3
Second coordination shell				
Sγ-HN (i+2) distance (Å)	2.28 ± 0.11	4.57 ± 0.91	2.77 ± 0.23	6.39 ± 0.37
Sγ-H-N (i+2) angle (º)	145.5 ± 3.8	131.3 ± 6.7	151.1 ± 7.7	116.2 ± 2.3

Table S3 (Related to STAR Methods). Plasmids used in this study.

Plasmid	Description[a,b]	Source
***For expression in/purification from* E. coli**		
pET-46-CsfB	*6xHis-CsfB lacI amp*	This study
pNIC28-CsfB	*6xHis-TEV-CsfB lacI kan*	This study
pNIC28-CsfB^{A48P}	*6xHis-TEV-CsfB*^{A48P} *lacI kan*	This study
pNIC28-CsfB^{A48L}	*6xHis-TEV-CsfB*^{A48L} *lacI kan*	This study
pNIC28-CsfB^{A48E}	*6xHis-TEV-CsfB*^{A48E} *lacI kan*	This study
pNIC28-CsfB^{F49A}	*6xHis-TEV-CsfB*^{F49A} *lacI kan*	This study
pNIC28-CsfB^{F49L}	*6xHis-TEV-CsfB*^{F49L} *lacI kan*	This study
pNIC28-CsfB^{Y50F}	*6xHis-TEV-CsfB*^{Y50F} *lacI kan*	This study
pNIC28-CsfB^{Y50E}	*6xHis-TEV-CsfB*^{Y50E} *lacI kan*	This study
pNIC28-CsfB^{K52A}	*6xHis-TEV-CsfB*^{K52A} *lacI kan*	This study
pNIC28-CsfB^{K53A}	*6xHis-TEV-CsfB*^{K53A} *lacI kan*	This study
pNIC28-CsfB^{K55A}	*6xHis-TEV-CsfB*^{K55A} *lacI kan*	This study
pLATE31-CsfB	*CsfB-6xHis lacI amp*	This study
pET-46-SigG	*6xHis-sigG lacI amp*	This study
pET28-TxrA-SigE	*6xHis-Trx-TEV-sigE*$^{17\text{-}239}$ *lacI kan*	This study
***For expression in/modification of* B. subtilis**		
pJJ46	*amyE*::P$_{spank}$-*sigG lacI spc, amp*	This study
pSFO1	*amyE*::P$_{hyperspank}$-*sigE*$^{17\text{-}239}$ *lacI spc, amp*	This study
pAH88	*thrC*::P$_{hyperspank}$-*csfB lacI erm, amp*	This study
pKF70	*thrC*::P$_{hyperspank}$-*csfB*^{A48E} *lacI erm, amp*	This study
pKF87	*thrC*::P$_{hyperspank}$-*csfB*^{V37A} *lacI erm, amp*	This study
pKF88	*thrC*::P$_{hyperspank}$-*csfB*^{V37E} *lacI erm, amp*	This study
pKF89	*thrC*::P$_{hyperspank}$-*csfB*^{I38A} *lacI erm, amp*	This study
pKF90	*thrC*::P$_{hyperspank}$-*csfB*^{I38E} *lacI erm, amp*	This study
pKF91	*thrC*::P$_{hyperspank}$-*csfB*^{V37A,I38A} *lacI erm, amp*	This study
pTK2	*thrC*::P$_{hyperspank}$-*csfB*$^{1\text{-}48}$ *lacI erm, amp*	This study
pAH334	*sacA*::P$_{spoIID}$-*luxABCDE cat*, *amp*	This study
pAH336	*sacA*::P$_{sspB}$-*luxABCDE cat*, *amp*	This study

[a]Antibiotic resistance genes are referred to as follows: *amp* (ampicillin), *cat* (chloramphenicol), *erm* (erythromycin plus lincomycin), and *kan* (kanamycin).

[b]See *Plasmid construction* section of STAR Methods for details.

Table S4 (Related to STAR Methods). Oligonucleotides used in this study.

**Oligonucleotide**	**Sequence (5’ → 3’)***
CsfB pET46 Fw	gacgacgacaagatggacgaaacagttaaac
CsfB pET46 Rv	gaggagaagcccggttatgaatataatggcggtg
CsfB pLATE31 Fw	agaaggagatataactatggacgaaacagtt
CsfB pLATE31 Rv	gtggtggtgatggtgatggcctgaatataatggcgg
CsfB *1-59* pNIC28 Fw	tacttccaatccatggacgaaacagttaaac
CsfB *1-59* pNIC28 Rv	tatccacctttactgtcatgtatgaatgctctttag
CsfB A48P mut Fw	catcaacttctgatcctgactatCcgttttacgtaaaaaaactaaagagcattc
CsfB A48P mut Rv	gaatgctctttagtttttttacgtaaaacgGatagtcaggatcagaagttgatg
CsfB A48L mut Fw	catcaacttctgatcctgactatTTgttttacgtaaaaaaactaaagagcattcatacac
CsfB A48L mut Rv	gtgtatgaatgctctttagtttttttacgtaaaacAAatagtcaggatcagaagttgatg
CsfB A48E mut Fw	cttctgatcctgactatgAgttttacgtaaaaaaactaaagagcattccatacacc
CsfB A48E mut Rv	ggtgtatggaatgctctttagtttttttacgtaaaacTcatagtcaggatcagaag
CsfB F49A mut Fw	caacttctgatcctgactatgcgGCttacgtaaaaaaactaaagagc
CsfB F49A mut Rv	gctctttagtttttttacgtaaGCcgcatagtcaggatcagaagttg
CsfB F49L mut Fw	catcaacttctgatcctgactatgcgCtttacgtaaaaaaactaaagagc
CsfB F49L mut Rv	gctctttagtttttttacgtaaaGcgcatagtcaggatcagaagttgatg
CsfB Y50F mut Fw	ctacatcaacttctgatcctgactatgcgttttTcgtaaaaaaactaaagagc
CsfB Y50F mut Rv	gctctttagtttttttacgAaaaacgcatagtcaggatcagaagttgatgtag
CsfB Y50E mut Fw	catcaacttctgatcctgactatgcgtttGaGgtaaaaaaactaaagagc
CsfB Y50E mut Rv	gctctttagtttttttacCtCaaacgcatagtcaggatcagaagttgatg
CsfB K52A mut Fw	cgttttacgtaGCaaaactaaagagcattcatacaccgcc
CsfB K52A mut Rv	ggcggtgtatgaatgctctttagttttGCtacgtaaaacg
CsfB K53A mut Fw	cgttttacgtaaaaGCactaaagagcattcatacaccgcc
CsfB K53A mut Rv	ggcggtgtatgaatgctctttagtGCttttacgtaaaacg
CsfB K55A mut Fw	cgttttacgtaaaaaaactaGCAagcattcatacaccgccattatattc
CsfB K55A mut Rv	gaatataatggcggtgtatgaatgcttGCtagtttttttacgtaaaacg
SigG pET46 Fw	gacgacgacaagatgtcgagaaataaagtcg
SigG pET46 Rv	gaggagaagcccggttattgatgaatatttttattc
SigE *17-239* pET28 Fw	cgcggatccatgaaactgggcctgaaaagtga
SigE *17-239* pET28 Rv	ttgcctcgagttacaccattttgttaaattctttgcgca
JJ30 (*sigG* Fw)	*gtgagcggataacaattaagcttagtcgac*gtacagcagctcctgtag
JJ31 (*sigG* Rv)	*ccgaattagcttgcatgcggctagc*ttattgatgaatatttttattcatttgtttgatag

continued →

**Oligonucleotide**	**Sequence (5’ → 3’)***
← *continued*	
AH41 (*csfB* Fw HindIII)	gatcaagctttacggaggtggagaagatg
AH42 (*csfB* Rv NheI)	gatcgctagctctactacgttcaatccttaaac
AH43 (Δ*sigE* P1)	aaatctatttagatgtcatttggctg
AH44 (Δ*sigE* P2)	*caattcgccctatagtgagtcgt*catcttcctctcccttctaaatg
AH45 (Δ*sigE* P3)	*ccagcttttgttccctttagtgag*taaaaaattttatggttagaacccccttg
AH46 (Δ*sigE* P4)	ccaaaacgtaaaccatccataatc
AH58 (P_{spoIID} Fw EcoRI)	gatcgaattcgatgagtctgcttctgagcaag
AH59 (P_{spoIID} Rv SalI)	gatcgtcgactgctcgggattcgactctag
AH60 (P_{sspB} Fw EcoRI)	gatcgaattcacgagatacatgaactgatgc
AH61 (P_{sspB} Rv SalI)	gatcgtcgactttttatttagtatggttgggttaactg
AH310 (MCS linker Fw)	aattcacagagctctcggcggccgcataactagtaagg
AH311 (MCS linker Rv)	tcgaccttactagttatgcggccgccgagagctctgtg
AH312 (BamHI mut Fw)	gcaatttctctgtcttaaagAatcctgaggaggaaaacagg
AH313 (BamHI mut Rv)	cctgttttcctcctcaggatTctttaagacagagaaattgc

*Restriction sites are underlined, regions matching plasmid sequences are italicized, and mutations are in uppercase

Table S5 (Related to STAR Methods). *B. subtilis* strains used in this study

Strain	Genotype[a,b,c]	Source
Strains for* in vivo *σ^G-functionality tests		
KF286	*ΔsigG*::*kan* Δ*csfB*::*tet* *sacA*::P$_{sspB}$-*luxABCDE cat*	This study
KF287	*ΔsigG*::*kan* Δ*csfB*::*tet* *sacA*::P$_{sspB}$-*luxABCDE cat* *amyE*::P$_{spank}$-*sigG lacI spc*	This study
KF288	*ΔsigG*::*kan* Δ*csfB*::*tet* *sacA*::P$_{sspB}$-*luxABCDE cat* *amyE*::P$_{spank}$-*sigG lacI spc* *thrC*::P$_{spank}$-*csfB lacI erm*	This study
KF386	*ΔsigG*::*kan* Δ*csfB*::*tet* *sacA*::P$_{sspB}$-*luxABCDE cat* *amyE*::P$_{spank}$-*sigG lacI spc* *thrC*::P$_{spank}$-*csfB*$^{1-48}$ *lacI erm*	This study
KF289	*ΔsigG*::*kan* Δ*csfB*::*tet* *sacA*::P$_{sspB}$-*luxABCDE cat* *amyE*::P$_{spank}$-*sigG lacI spc* *thrC*::P$_{spank}$-*csfB*^{A48E} *lacI erm*	This study
SFB9	*ΔsigG*::*kan* Δ*csfB*::*tet* *sacA*::P$_{sspB}$-*luxABCDE cat* *amyE*::P$_{spank}$-*sigG lacI spc* *thrC*::P$_{spank}$-*csfB*^{V37A} *lacI erm*	This study
SFB11	*ΔsigG*::*kan* Δ*csfB*::*tet* *sacA*::P$_{sspB}$-*luxABCDE cat* *amyE*::P$_{spank}$-*sigG lacI spc* *thrC*::P$_{spank}$-*csfB*^{V37E} *lacI erm*	This study
SFB15	*ΔsigG*::*kan* Δ*csfB*::*tet* *sacA*::P$_{sspB}$-*luxABCDE cat* *amyE*::P$_{spank}$-*sigG lacI spc* *thrC*::P$_{spank}$-*csfB*^{I38A} *lacI erm*	This study
SFB13	*ΔsigG*::*kan* Δ*csfB*::*tet* *sacA*::P$_{sspB}$-*luxABCDE cat* *amyE*::P$_{spank}$-*sigG lacI spc* *thrC*::P$_{spank}$-*csfB*^{I38E} *lacI erm*	This study
SFB17	*ΔsigG*::*kan* Δ*csfB*::*tet* *sacA*::P$_{sspB}$-*luxABCDE cat* *amyE*::P$_{spank}$-*sigG lacI spc* *thrC*::P$_{spank}$-*csfB*^{V37A,I38A} *lacI erm*	This study
Strains for* in vivo *σ^E-functionality tests		
SFB42	*ΔsigE*::*(erm)*::*phleo* Δ*csfB*::*tet* *sacA*::P$_{spoIID}$-*luxABCDE cat*	This study
SFB33	*ΔsigE*::*(erm)*::*phleo* Δ*csfB*::*tet* *sacA*::P$_{spoIID}$-*luxABCDE cat* *amyE*::P$_{hyperspank}$-*sigE*$^{17-end}$ *lacI spc*	This study
SFB38	*ΔsigE*::*(erm)*::*phleo* Δ*csfB*::*tet* *sacA*::P$_{spoIID}$-*luxABCDE cat* *amyE*::P$_{hyperspank}$-*sigE*$^{17-end}$ *lacI spc* *thrC*::P$_{spank}$-*csfB lacI erm*	This study
KCB32	*ΔsigE*::*(erm)*::*phleo* Δ*csfB*::*tet* *sacA*::P$_{spoIID}$-*luxABCDE cat* *amyE*::P$_{hyperspank}$-*sigE*$^{17-end}$ *lacI spc* *thrC*::P$_{spank}$-*csfB*$^{1-48}$ *lacI erm*	This study
SFB44	*ΔsigE*::*(erm)*::*phleo* Δ*csfB*::*tet* *sacA*::P$_{spoIID}$-*luxABCDE cat* *amyE*::P$_{hyperspank}$-*sigE*$^{17-end}$ *lacI spc* *thrC*::P$_{spank}$-*csfB*^{A48E} *lacI erm*	This study
SFB52	*ΔsigE*::*(erm)*::*phleo* Δ*csfB*::*tet* *sacA*::P$_{spoIID}$-*luxABCDE cat* *amyE*::P$_{hyperspank}$-*sigE*$^{17-end}$ *lacI spc* *thrC*::P$_{spank}$-*csfB*^{V37A} *lacI erm*	This study
SFB50	*ΔsigE*::*(erm)*::*phleo* Δ*csfB*::*tet* *sacA*::P$_{spoIID}$-*luxABCDE cat* *amyE*::P$_{hyperspank}$-*sigE*$^{17-end}$ *lacI spc* *thrC*::P$_{spank}$-*csfB*^{V37E} *lacI erm*	This study

continued →

Strain	Genotype[a,b,c]	Source
← *continued*		
SFB54	*ΔsigE*::*(erm)*::*phleo* Δ*csfB*::*tet* *sacA*::P_{spoIID}-*luxABCDE cat amyE*::$P_{hyperspank}$-*sigE*$^{17\text{-}end}$ *lacI spc* *thrC*::P_{spank}-*csfB*^{I38A} *lacI erm*	This study
SFB56	*ΔsigE*::*(erm)*::*phleo* Δ*csfB*::*tet* *sacA*::P_{spoIID}-*luxABCDE cat amyE*::$P_{hyperspank}$-*sigE*$^{17\text{-}end}$ *lacI spc* *thrC*::P_{spank}-*csfB*^{I38E} *lacI erm*	This study
SFB58	*ΔsigE*::*(erm)*::*phleo* Δ*csfB*::*tet* *sacA*::P_{spoIID}-*luxABCDE cat amyE*::$P_{hyperspank}$-*sigE*$^{17\text{-}end}$ *lacI spc* *thrC*::P_{spank}-*csfB*^{V37A,I38A} *lacI erm*	This study

[a]All strains are isogenic with the prototrophic wild type strain PY79 (Youngman et al., 1984)

[b]Antibiotic resistance genes are referred to as follows: *cat* (chloramphenicol), *erm* (erythromycin plus lincomycin), *kan* (kanamycin), *phleo* (phleomycin), *spc* (spectinomycin), and *tet* (tetracycline).

[c]For information on the sources of gene deletions, reporter genes, and other constructs, see *Plasmid construction* and *Strain construction* sections of the STAR Methods for details.
